# Recent Progress in Modifications, Properties, and Practical Applications of Glass Fiber

**DOI:** 10.3390/molecules28062466

**Published:** 2023-03-08

**Authors:** Yawen Wu, Yangyang Song, Di Wu, Xiaowei Mao, Xiuling Yang, Shaohua Jiang, Chunmei Zhang, Rui Guo

**Affiliations:** 1 Hubei Key Laboratory of Environmental and Health Effects of Persistent Toxic Substances, School of Environment and Health, Jianghan University, Wuhan 430056, China; ruanquwei327@163.com (Y.W.); 18406573975@163.com (Y.S.); wu2021di@163.com (D.W.); maoxiaowei5167@126.com (X.M.); 2 Jiangsu Co-Innovation Center of Efficient Processing and Utilization of Forest Resources, International Innovation Center for Forest Chemicals and Materials, College of Materials Science and Engineering, Nanjing Forestry University, Nanjing 210037, China; xiuling_yang_lab@126.com; 3 Institute of Materials Science and Devices, School of Materials Science and Engineering, Suzhou University of Science and Technology, Suzhou 215009, China

**Keywords:** modified glass fibers, multiple modification strategies, performance properties, advanced applications

## Abstract

As a new member of the silica-derivative family, modified glass fiber (MGF) has attracted extensive attention because of its excellent properties and potential applications. Surface modification of glass fiber (GF) greatly changes its performance, resulting in a series of changes to its surface structure, wettability, electrical properties, mechanical properties, and stability. This article summarizes the latest research progress in MGF, including the different modification methods, the various properties, and their advanced applications in different fields. Finally, the challenges and possible solutions were provided for future investigations of MGF.

## 1. Introduction

Glass fiber (GF) is a cotton-like material obtained by fibrosis of molten glass [1]. It is an inorganic, nonmetallic material with a number of excellent properties, such as a high aspect ratio, small volume density, low thermal conductivity, strong corrosion resistance, stable chemical performance, and electrical insulation [2,3,4,5,6,7]. It has become an important adsorbent in the field of chemistry and an important new, lightweight, and energy-saving composite multifunctional carrier. It can be used for insulation [8], fire prevention [9,10], heat insulation [11,12,13], sound insulation [14,15,16], adsorption [17,18], and as a polymer precursor [19,20,21]. It is widely used in plastics, rubber, chemicals, aerospace, and other fields. However, it still has some disadvantages, such as brittleness and poor wear resistance, which hinder the direct application of GF. A variety of physical and chemical modification methods have been developed to modify the GF surface before application to solve these problems and improve its compatibility and binding ability with the polymer matrix by changing the structural composition of the GF surface [22].

The main component of GF is amorphous silicon dioxide, which exists in the form of a polymer (SiO_2_). The silicon atoms form a three-dimensional network structure by corner-sharing oxygen atoms [23,24,25]. The reticular structure determines that GF is a highly inert material, and thus its surface must be functionalized to produce modified GF (MGF) and expand the application range of GF.

In this paper, we summarize the latest research progress in MGF from the following three aspects: (I) The surface modification methods of GF, including coating modification, grafting modification, and fluorination modification; (II) the characteristics of MGF, including the changes in the surface structure and mechanochemical properties upon modification; and (III) the application scope of MGF, including as an adsorbent, in composite reinforcement materials, in the biomedical field, and in other fields. In addition, on the basis of the existing achievements, we give our own views and the prospects for solving the existing problems and overcoming the challenges in this field. We hope that this review of MGF from synthesis to application, and in particular the critical review of the modification methods and properties, will provide guidance for targeted and precise modification of other materials.

## 2. Surface Modification Methods

Surface modification is an important deep-processing method for GF. It involves physical, chemical, or mechanical treatment of the GF surface according to the requirements to improve the surface activity of the GF or improve the compatibility of the GF with polymers. Surface modification is based on the existence of active groups, such as Si–O and Si–OH bonds. It is characterized by only changing the composition of the interface layer of the GF without changing the internal structure and physical and chemical properties, even if the chemical reaction method is used.

**Figure 1:**
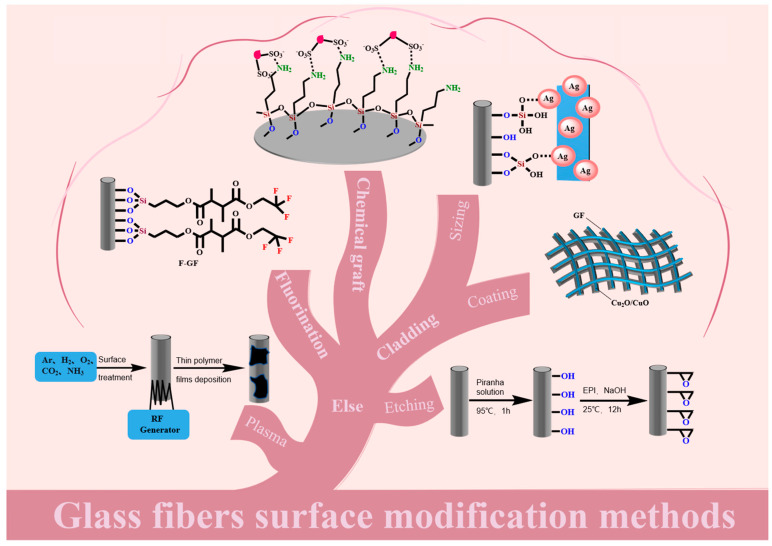
Glass fibers surface modification methods (by Figdraw).

There are a variety of surface modification methods for GF, as described in Figure 1. The most common methods are coating modification [26,27,28,29,30], fluorination modification [13], chemical grafting modification [31,32,33], redox modification [22], acid–base etching modification [34,35,36], plasma treatment [37,38], and doping modification [39,40]. These modification methods can improve the comprehensive properties of GF and provide a way for the functionalization or even multi-functionalization of various GF derivatives.

### 2.1. Coating Modification

Surface-coating methods are methods in which an organic, metal, or nonmetal coating is coated on the GF surface by chemical bonding, physical deposition, or chemical adsorption. Different coatings have different properties, and coating modification shows versatility in modifying the GF-surface properties. Customized coating methods [41,42], such as dip coating, the spraying method, electroplating [43,44,45,46,47,48], pyrolysis [49,50,51], electrophoretic deposition [52,53,54], and electrothermal shock [55], are currently used to modify GF.

#### 2.1.1. Sizing Modification

A promising modification method for GF is fiber sizing, in which the fibers are coated with a slurry mainly consisting of film formers and coupling agents. In addition to these two major components, the slurry may include additional lubricants, antistatic agents, and surfactants [56,57,58,59]. A complex sizing formula may contain ten or more components.

The advantages of sizing modification are the production of composite products, cost-effective and efficient manufacturing, optimized adhesion to the substrate interface, increased short- and long-term performance of the composite products, protection of the fiber surface from damage and environmental degradation, reduction of entry of moisture, and the added function of the interface region.

##### Film-Forming-Agent Modification

In slurry formulations, the film formers usually make up 70–90% of the material. The film formers form high-molecular-weight and water-insoluble materials on the GF surface by emulsion dispersion technology to protect, fix, and lubricate the GF and promote separation from the matrix during subsequent fiber processing. Commonly used film formers include polyvinyl acetate, polyurethane, polyolefin, polyester, epoxy resin, and modified epoxy resin [60].

**Figure 2:**
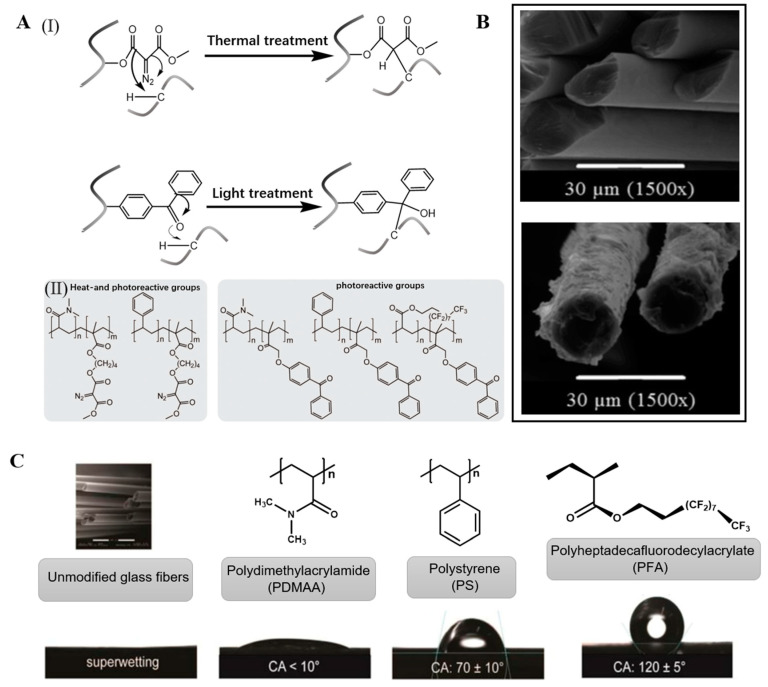
(**A**) (**I**) Schematic description of the mechanism of chemical modification of the fiber-matrix interface using adhesion accelerators. (**II**) Polymer systems selected as adhesion promoters containing diazacarbonyl (**left**) and benzophenone groups (**right**). (**B**) SEM of fiber-matrix interface. (**C**) Tailoring the wetting behavior of glass fibers upon coating with hydrophilic polymers and hydrophobic polymers. Reproduced with permission from reference [61]. Copyright 2019 *Advanced Engineering Materials*.

Abdul et al. used the dip-coating method to physically deposit polymer films containing photoreactive dibenzophenone residues or diazocarboxyl groups (Figure 2A(II)) on the GF surface. After activation by light and heat stimulation, the formed reactive intermediates crosslinked by adjacent C–H insertion, as shown in (Figure 2A(I)). The surface was coated with a strong crosslinked polymer network. The presence of the coating at the fiber surface could be visualized using scanning electron microscopy (Figure 2B), which reveals the glass fiber topography before and after modification with two different PS-based polymers. The polymer was covalently connected to the fiber surface. This type of sizing agent is nonspecific, widely applicable, and reusable [61].

##### Silane-Coupling-Agent Modification

**Figure 3:**
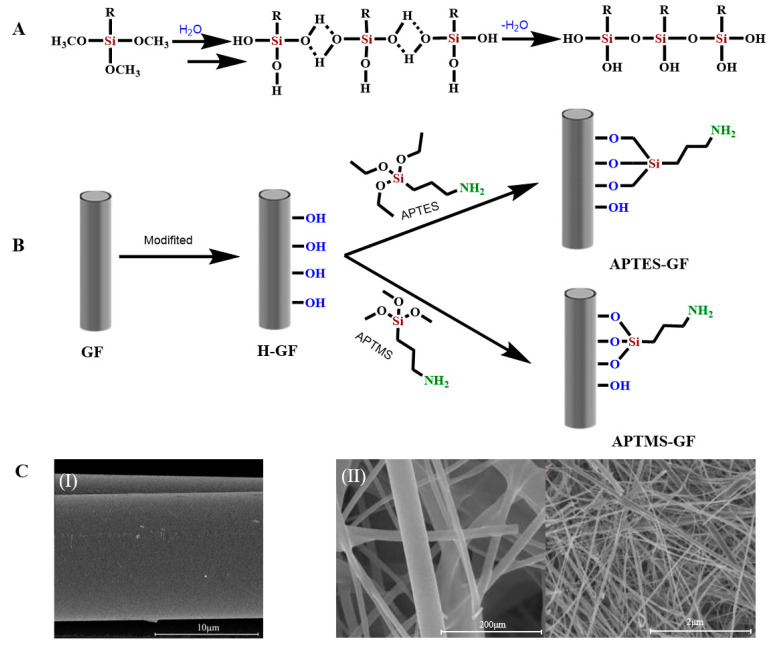
(**A**) The schematic of the self−condensation reaction of silane-coupling agent. (**B**) Schematic diagram of a modification of glass fiber by an amino-silane coupling agent. (**C**) SEM morphology of (**I**) APTES−GF. Reproduced with permission from reference [62]. Copyright 2014 *Composites Science and Technology Society of Chemistry*. (**II**) APTMS–GF. Reproduced with permission from reference [64]. Copyright 2018 *ChemistrySelect*.

Another main component of the slurry is the silane coupling agent. Silane-coupling agents include a variety of different organosilane molecules. Their general structure is [R–Si(OX)_3_], where X is a methyl or an ethyl group, and the R group contains amino, epoxy, methylacryloxy, or vinyl functional groups. One end of the molecule reacts with the GF surface, while the other end reacts with the polymer matrix [56]. The interaction between the silane-coupling agent and GF is usually described as a condensation reaction of surface silanols (Figure 3A). The silane coupling agent first hydrolyzes into unstable silanols, and the silanols and hydroxyl groups on the fiber surface coalesce by eliminating water molecules. The siloxane network is covalently bonded to the fiber surface. Because the silane-coupling agent shows the tendency of self-condensation, the finally formed siloxane has a low density and is easily hydrolyzed and unstable. When the fiber is subsequently applied to the polymer matrix, the X group of the silane is used to react with the reactive functional group of the polymer, forming a strong network bridging the fiber–polymer interface [62,63].

The four most common silane coupling agents are γ-aminopropyl-triethoxysilane (APTES) [65,66], γ-epoxypropyl-trimethoxysilane (GPTMS) [67], γ-methylpropyl-trimethoxy-silane (MPTMS) [27,63], and vinyltriethoxy-silane (VTES) [68].

**Figure 4:**
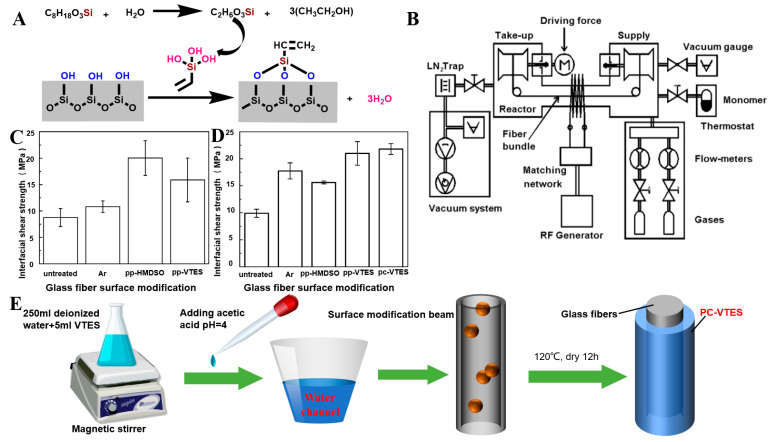
(**A**) Diagram of condensation reaction mechanism between silica alcohol and glass fiber interface. (**B**) Schematic diagram of plasma technology for continuous surface treatment and modification of fiber bundle. (**C**) Interfacial shear strength was determined from a microbond test for untreated, plasma-treated, and plasma polymer-coated glass fibers. (**D**) Short −beam strength of glass fiber/polyester composite for plasma-modified, wet-chemical modified, and untreated fibers. (**E**) Wet chemical preparation process. Reproduced with permission from reference [62]. Copyright 2013 *Journal of Adhesion Science and Technology*.

Cech et al. applied a wet chemical method (Figure 4A,E) to coat VTES in an aqueous solution on the surface of unmodified GF to form polycondensed VTES, and a siloxane oligomer layer formed on the GF surface. The ethoxyl group (–OCH_2_CH_3_) is a hydrolysis unit that condenses between the silanol/glass fiber interface and adjacent silanol molecules to form a polysiloxane layer and a series of outward-oriented vinylates (CH_2_=CH–) that are subsequently used as reinforcement for unsaturated resins [62]. Chen et al. used APTES to modify the GF surface. GF was immersed in APTES (NH_2_(CH_2_)_3_–Si(OCH_2_CH_3_)_3_) organic solution to fully hydrolyze APTES, as shown in (Figure 3B), forming APTES-modified GF [69,70]. Similarly, Li et al. modified a GF membrane (GFM) with 3-(trimethoxy-methylsilyl)-1-propylamine (NH_2_(CH_2_)_3_–Si(OCH_3_)_3_, APTMS) to obtain APTMS-modified GF [64].

#### 2.1.2. Mental Coating

In the past decade, metal coating materials have been widely investigated because of their good electrical conductivity and strong anti-interference ability, and they have been applied in many fields. Gold [71], silver [72,73,74], copper [48], cobalt [75], aluminum, and nickel [76,77] have been investigated as metal coatings for the GF surface. However, metal coatings also have disadvantages, such as poor flexibility, high density, and poor corrosion resistance. Thus, their practical application is limited.

**Figure 5:**
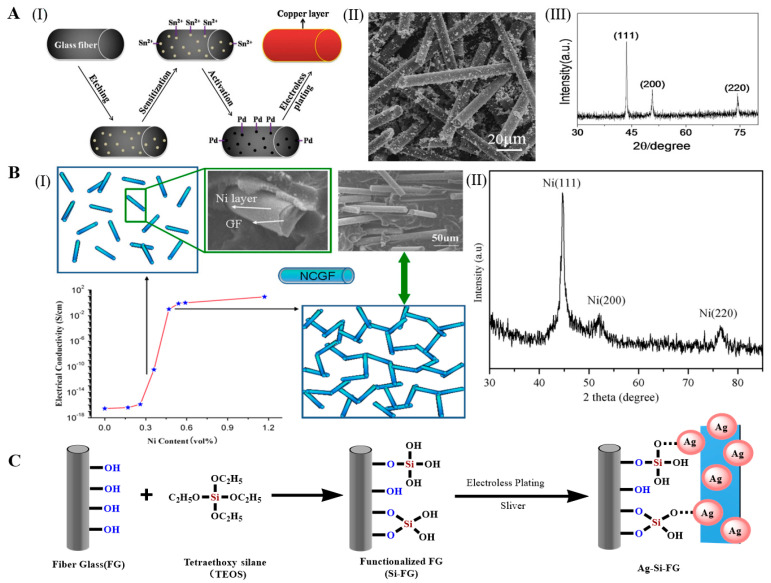
(**A**) Schematic (**I**) illustration for the electroless plating of copper on glass fibers. (**II**) SEM images of the as−obtained copper−coated glass fibers. (**III**) XRD pattern of the as−synthesized copper−coated glass fibers. Reproduced with permission from reference [45]. Copyright 2014 *Springer*. (**B**) schematic (**I**) for the formation of conductive paths in the composites and (**II**) XRD pattern for NCGFs. Reproduced with permission from reference [77]. Copyright 2015 *Elsevier*. (**C**) Reaction diagram of electroless silver plating on TEOS−modified FG.

Xu et al. prepared copper-coated GF by an autocatalytic process under optimized conditions. After etching, sensitization, and activation of the GF, the copper-coated GF was prepared by reducing Cu^2+^ under alkaline conditions using CuSO_4_ as the main salt, hydrazine hydrate (N_2_H_4_·H_2_O) or formaldehyde as the reducing agent, and ethylenediaminetetraacetic acid as the stabilizer (Figure 5A(I)). The copper layer on the GF surface was dense and uniform, and some copper particles were isolated from the fibers, and a small amount of them was attached to the surface of the fibers (Figure 5A(II)). In addition, through the XRD of a pattern of the product, the amorphous signals for glass fibers did not emerge, which indicated that a layer of copper was successfully deposited on the surface of the glass fibers via electroless plating. (Figure 5A(III)) [45,47,48]. Guo et al. successfully prepared a continuously conducting two-dimensional glass fiber network containing nickel by electroless plating, and SEM and XRD patterns showed a uniform and more reduced mental nickel coating deposited on the glass fiber substrate(Figure 5B(II)). The low percolation threshold and superior electrical conductivity were attributed to the high aspect ratio of glass fibers and uniform distribution of the Ni located at the interface between glass fibers and the PP matrix, respectively (Figure 5B(I)) [77]. Xu et al. used an autocatalytic process under optimized conditions to prepare silver-coated GF. Ag+ was reduced by placing the GF in an AgNO_3_ solution after sensitization and activation. The silver coating on the GF surface was dense and uniform [47]. However, the sensitization and activation processes were cumbersome and costly. Tetraethoxysilane (TEOS) modification technology has replaced traditional sensitization and activation. Lien et al. grafted TEOS on the GF surface and prepared silver-coated GF by strong adsorption of Ag+ on the TEOS-grafted GF (Figure 5C). The alkyl group improved the stability of the electroless silver-plated GF by forming a covalent bridge with silver [44]. Huang et al. deposited a layer of Ni–Co–P alloy on the GF surface by the electroless plating method, and they adjusted the composition of the Ni–Co–P alloy and electroless plating rate by changing the electroless plating parameters (e.g., the CoSO_4_/(CoSO_4_ + NiSO_4_) molar ratio and pH). By increasing the CoSO_4_/(CoSO_4_ + NiSO_4_) molar ratio and pH, the element content changed in the same direction, the cobalt content increased, and the nickel and phosphorus contents decreased, but the chemical rate changed in the opposite direction. The electroless plating rate decreased with increasing CoSO_4_/(CoSO_4_ + NiSO_4_) molar ratio, but it increased with increasing pH. The electroless plating rate was the highest when the pH was 9.0 [43].

#### 2.1.3. Metal-Oxide Coating

A thin film can be formed on the fiber surface by dipping the metal oxide on the fiber base [78]. Incorporating different metal phases into the fiber network and forming a hybrid metal oxide coating can improve the mechanical properties of the fiber to a varying degree. Liu et al. impregnated GF with mixed solutions of Mn(NO_3_)_2_ and Ce(NO_3_)_3_·6H_2_O in different proportions to prepare a series of Ce/Mn-modified GF composites with different contents. The results showed that CeO_2_ and MnOx were uniformly loaded on the GF surface in an amorphous form [79]. Avila-Lopez et al. used the microwave hydrothermal method to prepare CuO coatings on three different GFs. They immersed GF in an aqueous solution of Cu(CH_3_COO)_2_·H_2_O and microwave irradiated the solution at 80 °C for 1 h. CuO was deposited on the three different GFs, and the CuO coating showed different forms in the different GFs, namely, strips and spheres [80]. Sayaka and Aminian et al. prepared a TiO_2_ porous GF composite by infiltrating GF into a TiO_2_ mixture using the traditional dip-coating method, and TiO_2_ was dispersed on the GF surface in the form of anatase particles [81,82,83]. Gutierrez et al. coated GF with hematite nanoparticles (ferric oxide particles). First, GF was immersed in FeCl_3_ solution, and it was then immersed in 15% NH_4_OH solution and decomposed with heat. The final FeO load was 25%, and FeO covered most of the exposed surface of the GF to form a layer with uneven thickness [84].

#### 2.1.4. Non-Metallic Coating

Non-metallic coating of GF overcomes the disadvantages of the high density and poor corrosion resistance of metal-coated GF. Potential candidates for non-metallic coating of GF include graphene [85], graphene oxide (GO) [86], reduced GO (RGO) [87], carbon nanotubes (CNTs) [53,55], and boron nitride nanotubes (BNNTs) [88]. All of these materials have been used to produce GF coatings, but the electrical conductivities of the coated GFs were slightly lower than those of metal-coated GFs.

**Figure 6:**
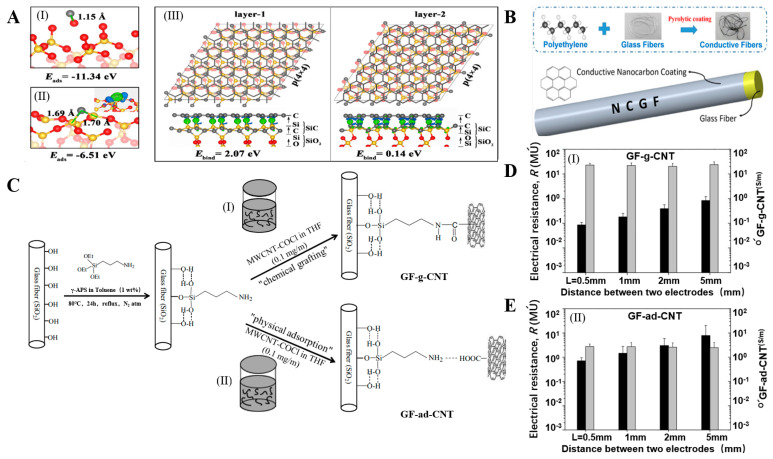
(**A**) (**I**) Initial adsorption of carbon atoms on the SiO_2_ (001) surface. Red, golden, and gray balls represent O, Si, and C atoms, respectively. (**II**) Adsorption of carbon atoms after the partial deoxidation of the SiO_2_ (001) surface and its corresponding charge difference maps (shown in the inset). (**III**) Two kinds of optimized graphene/SiC/SiO_2_ structures and the corresponding charge difference maps of the interface between the graphene−carbon layer and the SiC/SiO_2_ substrate. Blue and green regions are on behalf of electron depletion and accumulation, respectively. (**B**) Diagram of conductive nano carbon coated glass fiber. Reproduced with permission from reference [51]. Copyright 2020 *The Journal of Physical Chemistry C*. (**C**) Illustration of (**I**) MWCNTs chemically grafted (GF−g−CNT) and (II) physically adsorbed (GF−ad−CNT) to the GF surface. A dip−coating deposition process was used in both cases (a color version of this figure can be viewed online). DC electrical resistance (R, black bars) and conductivity (r, grey bars) of single GFs shown with (**D**) chemically grafted (GF−g−CNT) and (**E**) physically adsorbed (GF−ad−CNT) MWCNTs as a function of the electrode distance (error bars represent the corresponding standard deviations). Reproduced with permission from reference [70]. Copyright 2014 *Carbon*.

**Figure 7:**
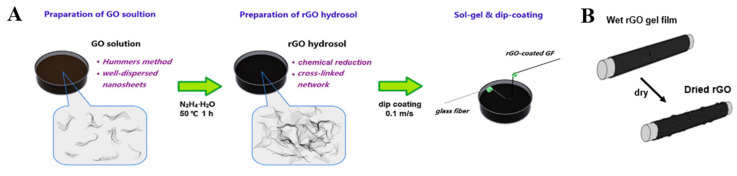
(**A**) Preparation schematic of graphene−coated GFs through sol-gel and dip−coating techniques. (**B**) Formation of the wrinkled rGO coatings onto GFs. Reproduced with permission from reference [87]. Copyright 2019 *Journal of Materials Science & Technology*.

**Figure 8:**
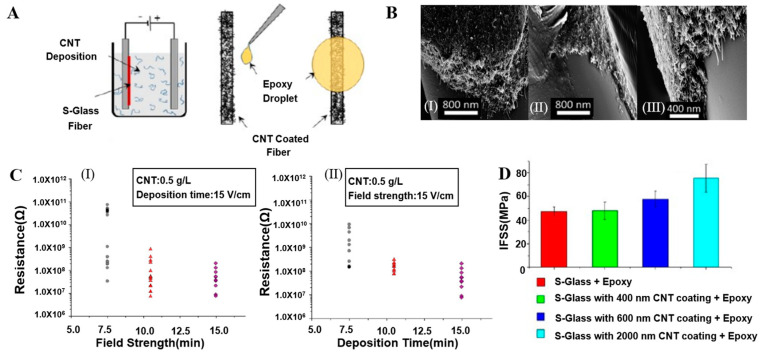
(**A**) The coating thickness of carbon nanotubes on glass fiber was controlled by electrophoretic deposition to adjust the interface properties. (**B**) Wettability between CNT and epoxy: (**I**) SG−1−15−15 m, (**II**) SG−15−10.5 m, and (**III**) SG−CNT/EP−CNT. (**C**) Electrical resistance measurement of EPD fibers at the CNT concentration 0.5 g/L: (**I**) at different field strengths with constant deposition time of 15 min and (**II**) at different deposition times with constant field strength of 15 V/cm. (**D**) Interfacial shear strength of different EPD fiber and epoxy systems. Reproduced with permission from reference [95]. Copyright 2016 *ACS Applied Materials & Interfaces*.

Alcaniz-Monge, Cheng, and Gao et al. generated and deposited free active carbon atoms on GF through pyrolysis of polyethylene, asphalt, and methane, respectively, forming a graphene/GF linear structure, as shown in Figure 6A,B. The deposited nano carbon showed good arrangement symmetry. The surface of the GF was uniformly and continuously coated by graphene lamination, forming a dense structure [49,50,51]. Jing et al. mixed GF modified by a silane-coupling agent (S-GF) and GO aqueous solution to obtain GO-coated GF (GO-GF) by an electrostatic assembly strategy. Compared with the relatively smooth surface of the S-GF, the surface of the GO-GF was wrinkled and rougher, indicating that GO was successfully coated on the GF surface. Raman spectroscopy confirmed the existence of GO [89,90]. Mahmood et al. deposited negatively charged GO particles on positively charged GF by triboelectrification. A chemically reduced GO coating was then generated to obtain an RGO layer on the GF surface, which completely adhered to the GF surface [41]. Fang et al. prepared RGO-coated GF using sol–gel and dip-coating technology (Figure 7A). After repeated dip coating and cyclic drying and dehydration (Figure 7B), multiple layers of RGO formed a densely interconnected network, completely covering the GF surface [87,91]. Siyal et al. immersed a GFM in polydimethylsiloxane (PDMS) dispersions with different concentrations of fluoromethane (FG). The characteristic peaks of the C–F band at 1212 cm^−1^ and FG at 1220 cm^−1^ in the Fourier transform infrared spectrum indicated that surface modification of the coating film was successful. Scanning electron microscopy (SEM) results showed that with increasing FG content, the FG distribution density on the GFM surface increased, as did the irregularity of the film [92]. Moriche et al. deposited NH_2_-functionalized graphene nanoplatelets (NH_2_-GNPs) on the GF surface, resulting in a strong interaction between the NH_2_-GNPs and GF, improving the electrical and mechanical properties of the GF [93]. Ye et al. introduced multi-walled CNTs on the GF surface with the aid of the sodium dodecylbenzenesulfonate surfactant. The CNTs formed a uniform network covering the GF surface [94]. Tamrakar et al. deposited polyethylenimine (PEI)-functionalized multi-walled CNT films on the GF surface by electrophoretic deposition (Figure 8A). The GF was covered with a thin layer of CNTs (Figure 8B). The film thickness was controlled in the range from 200 nm to 2 μm [95,96]. Takizawa immersed GF coated with epoxy resin in a toluene dispersion of BNNTs with a BNNT content of 0.021 wt%. The epoxy resin on the GF surface dissolved, and the BNNTs dispersed in toluene deposited on the GF surface [88]. Zhang introduced silica nanoparticles on the GF surface and coated the GF surface with a thin layer of SiO_2_ [97,98,99]. Parizi et al. modified the GF surface with three different silica coating films, namely, a silica microporous film, a mesoporous silica film, and a silica gel film. Through modification, the three SiO_2_ layers showed different pore sizes, size distributions, surface areas, and thicknesses [3].

#### 2.1.5. Composite Coating

In recent years, research on GF surface coating has gradually increased, and metal/metal oxide/nonmetal composite coatings have attracted wide attention and become a new research focus [100]. Glass fiber contains multiple organic and inorganic phases of composite coating modification, composite coating composition, and surface morphology is the biggest winner affecting the interface performance of glass fibers, because of its high permeability in glass fiber, composite coating fibers than other single-phase coating fibers have better material properties. It is also helpful for the development and application of fiber-reinforced composites.

**Figure 9:**
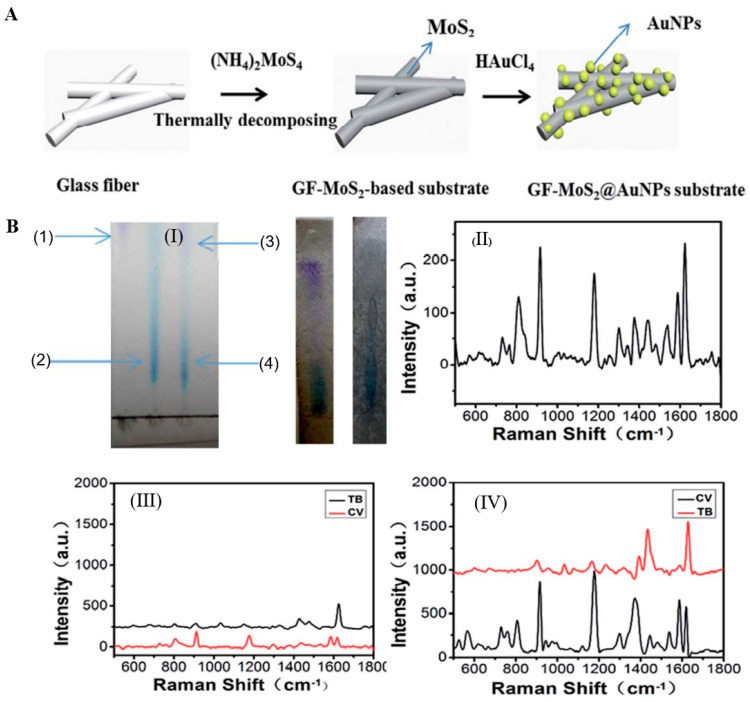
(**A**) Schematic of the synthesis of the GF−MoS_2_@AuNP substrate. (**B**) (**I**) Colorimetric detection of dye molecules (CV, TB, and their mixed solution) after paper separation and images of the mixed solution after separation on the MoS_2_ and GF-MoS_2_@AuNP−4 substrates. (**II**) Raman spectra of the mixed solution. Sub−figures (**III**) and (**IV**) are Raman spectra of the CV and TB molecules after paper separation on the MoS_2_ and GF−MoS_2_@AuNPs substrates. Reproduced with permission from reference [71]. Copyright 2017 *RSC Advances*.

Wei et al. uniformly loaded BiOI/BiOBr/MoS_2_ powder on the GF surface to form the BiOI/BiOBr/MoS_2_–GF composite material. BiOI/BiOBr/MoS_2_ was superimposed on the GF surface in a hexagonal-layered structure, providing active sites for subsequent photodegradation of ammonia nitrogen wastewater by the composite material [101]. Zhao et al. synthesized layered gold nanoparticle (AuNP)/MoS_2_/GF composites by cycling pyrolytically coated GF into tetrachloroauric acid (HAuCl_4_) (Figure 9A). The AuNPs were uniformly and extensively deposited on the unbound sulfur sites on the GF surface, and the activity of the surface-enhanced Raman scattering peak increased with an increasing number of cycles. The intensity of the Raman peak increased, and the AuNP/MoS_2_/GF Raman peak intensity was the highest (Figure 9B(II–IV)) [71]. Li et al. sprayed a GO/ZnO/Cu_2_O mixed coating on the surface of ultrafine GF and prepared the GO/ZnO/Cu_2_O–GF composite material. GO, ZnO, and Cu_2_O were loaded on the surface of the GF composite [100]. Gao et al. dispersed low-concentration CNTs in a film-forming agent (composed of ATTES and maleic-anhydride-grafted polypropylene) to make a CNT–film-forming-agent mixed nanocoating and then coated it on the GF surface. The coating repaired the surface defects of the GF and improved the strength, corrosion resistance, and interface performance of the GF [26]. Zhou et al. fabricated biomimetic polydopamine (PDA)-functionalized polytetrafluoroethylene (PTFE)/GF fabric using the sodium iodate oxidant to accelerate the polymerization of dopamine. PDA was successfully deposited on the surface of the PTFE/GF fabric, and the uniformity and thickness of the PDA coating increased with increasing modification time [102]. Meng et al. introduced montmorillonite modified with quaternary ammonium compounds (QACMt) into the PTFE/GF matrix to prepare the QACMt–PTFE/GF composite, which was a high-performance material with extremely strong antibacterial activity and mechanical properties [103].

### 2.2. Chemical Grafting Modification

The chemical grafting method introduces functional side groups by the free radical polymerization reaction of functional groups, such as –OH or –NH_2_ groups, on the GF surface, and a new layer of grafted polymer with special properties forms on the GF surface. At present, the investigation of graft polymers mainly focuses on organic functional groups and inorganic nanomaterials.

Silane-coupling-agent modification and film-forming-agent modification can also be classified as chemical grafting modification methods as pretreatment methods for grafting other polymers to provide active groups for the subsequent grafting reaction.

#### 2.2.1. Grafting Organic Functional Groups

The interfacial properties of the fibers are changed by grafting monomers of reactive organic functional groups onto the fiber surface through polymerization. Organic functional groups grafted on the GF surface include alkanes, perfluorinated chains, benzene, silane coupling agents, and film-forming agents. After the organic functional groups bond with the fiber, although the surface form of the glass fiber is comparatively richer and the reactivity is improved, the post-treatment process of the organic reagent is complicated. In particular, fluorinated reagents produce secondary pollution and pose a serious threat to the environment and human health.

**Figure 10:**
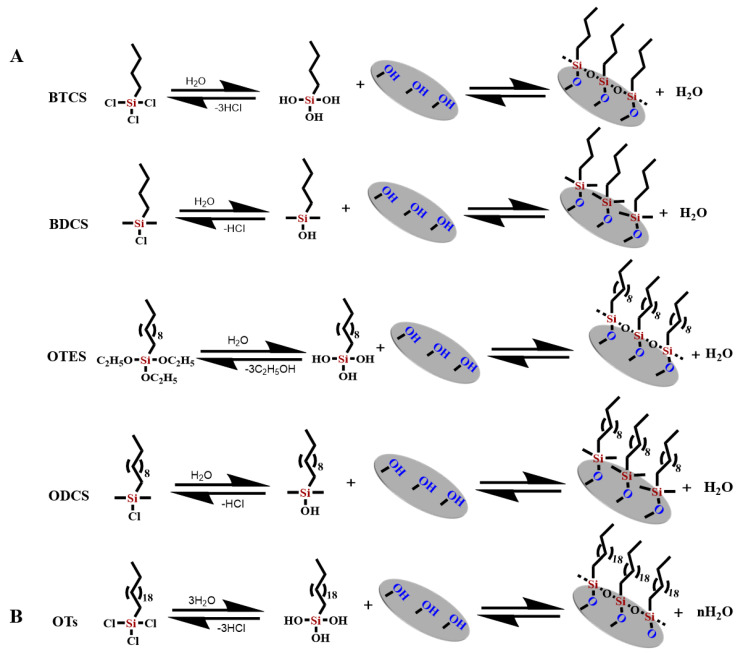
(**A**) Reaction schemes for modifications of glass fiber membrane with different organosilicon derivatives. (**B**) Reaction scheme of glass fiber membrane with octadecyl trichlorosilane.

**Figure 11:**
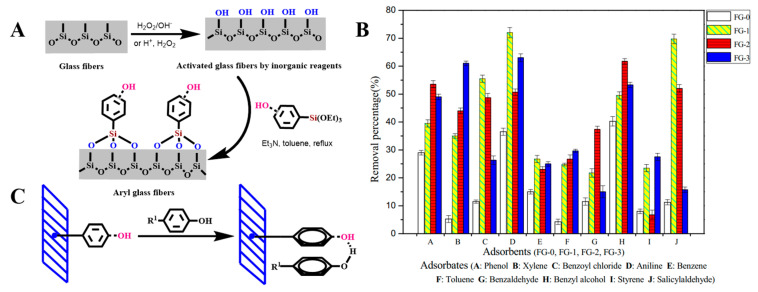
(**A**) Preparation of the activated glass fibers and aryl glass fibers. (**B**) The removal percentages for the four glass fiber types with regard to specific volatile benzene −based compounds (bars show standard deviations for *n* = 4); one of the FG −0 (untreated glass fibers), FG −1 (30% H_2_O_2_), FG −2 (NaOH + ethanol + distilled water) and FG −3 (98% H_2_SO_4_ + 30% H_2_O_2_). (**C**) A diagram showing the adsorption of aromatic compounds by the glass fibers via both hydrogen bonding and π–π conjugate interactions. Reproduced with permission from reference [22]. Copyright 2021 *Environmental Science and Pollution Research International*.

**Figure 12:**
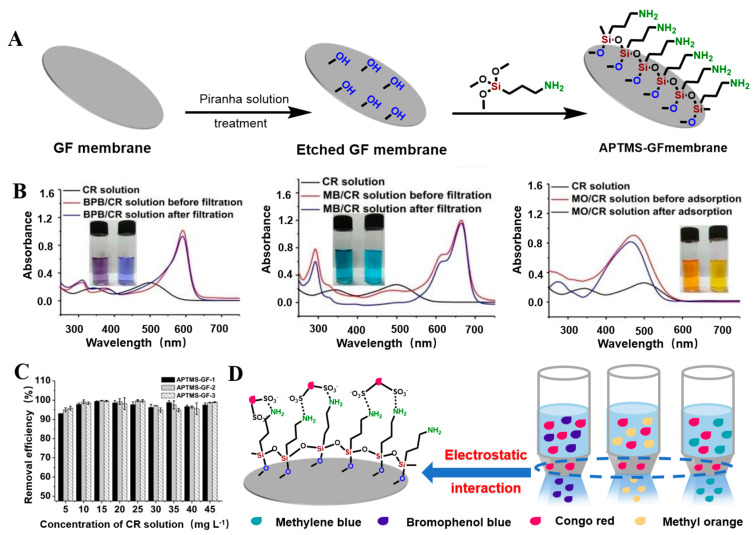
(**A**) Fabrication of the APTMS−GF composite membrane. (**B**) The UV−vis spectra of mixed dye solutions before and after filtration by the APTMS−GF membrane. (**C**) Removal efficiencies of a set of CR solutions with varied concentrations by APTMS−GF−1 membrane, APTMS–GF−2 membrane, and APTMS−GF−3 membrane, respectively. Reproduced with permission from reference [109]. Copyright 2018 *ChemistrySelect*. (**D**) Schematic of the proposed separation mechanism on the APTMS–GF composite membrane.

**Figure 13:**
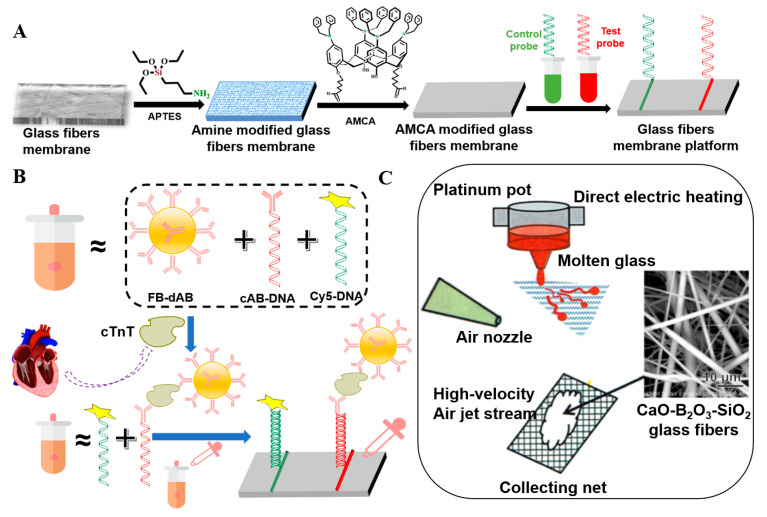
(**A**) DNA−modified glass fiber membranes by using the DAGON method. (**B**) cTnT was detected by antibody coupling with fluorescent beads. Reproduced with permission from reference [69]. Copyright © 2012 *The Analyst*. (**C**) Schematic representation of the preparation of glass fibers. Reproduced with permission from reference [113]. Copyright 2022 *Materials In Medicine*.

**Figure 14:**
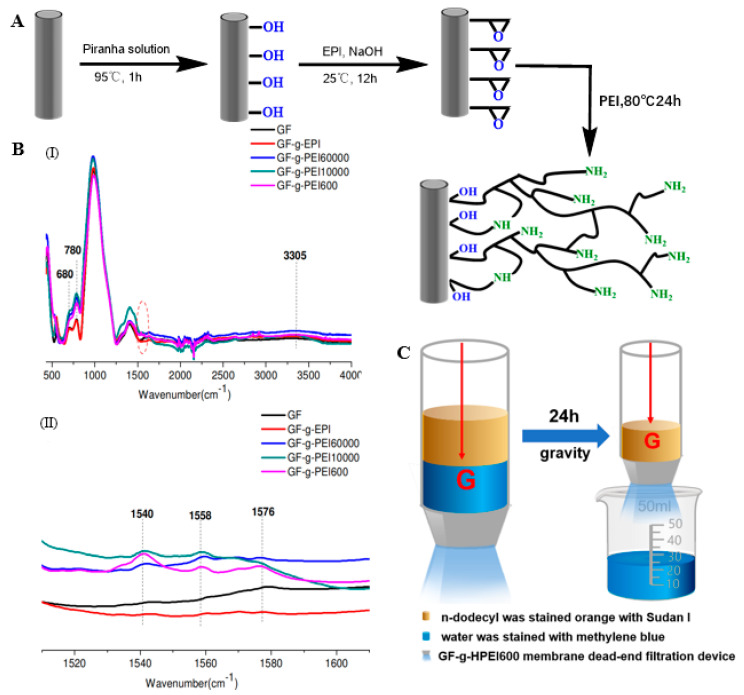
(**A**) Reaction mechanism of HPEI grafting onto glass fiber membrane surface. (**B**) ATR-FTIR spectra of GF, GF−g−EPI, GF−g−HPEI60000, GF−g−HPEI10000, and GF−g−HPEI600 membranes. (**I**) Full spectra from 500–4000 cm^−1^ and (**II**) high-resolution spectra from 1510–1610 cm^−1^. Reproduced with permission from reference [109]. Copyright 2017 *Chemical Engineering Journal*. (**C**) Images of the O/W separation apparatus equipped with the modified membrane.

Chen et al. modified porous GFMs with different chain lengths (C_4_, C_8_, and C_18_), including butyltrichloro-silane, butyldimethyl-chlorosilane, octyltriethoxy-silane, octyldimethylchloro-silane, and octyltrichloro-silane. The organosilicon derivatives successfully adhered to the surface of the modified GFM [104,105], as shown in Figure 10A,B. Guo et al. used a phenyl coupling agent to modify GF cotton chemically. The activated GF treated with piranha solution was further modified with the triethoxysilyl benzene compounds triethoxy(phenyl)silane, 4-(triethoxysilyl)phenol, and 4-(triethoxysilyl)benzene-1,2-diol to obtain aryl-modified GFs (Figure 11A). SEM results showed that the GF was successfully connected with the phenyl group, and the three aryl-modified GFs had larger surface areas and more porous structures than the original GF [22]. Equally, as shown in Figure 12A, Li et al. etched the glass fiber with piranha solution to increase the abundance of the silanol group and then chemically modified it with APTMS via building Si–O–Si bonds. The UV-vis spectra (Figure 12B) of the mixed solution before and after separation showed that the APTMS–GF membrane had the ability to selectively separate dyes, while the unmodified glass fiber membrane did not. Song et al. chemically modified a GFM by a two-step modification method (Figure 13A) [69]. First, the GFM was treated with APTES. The amine functional group reacted with aminocalixarene-1,3-dialdehyde (AMCA) to synthesize an AMCA-modified GFM [106,107,108]. Cao et al. grafted hyperbranched PEI (HPEI) on the surface of a GFM to synthesize a super hydrophilic GF–G–PEI film using ephedrine as the intermediate. The surface structure of the modified film was intact and coarse, with obvious granular nodules (Figure 14A). Fourier transform infrared spectroscopy results (Figure 14B) showed that HPEI molecules were successfully grafted on the GFM surface [109]. Li aniline on a GFM through a multi-step reaction, and they produced a smooth, uniform, and stable thin layer of polyaniline on the GF surface [110,111]. Gonzhlez-Benito et al. grafted a pyrene fluorescent probe on the GF substrate and 1-pyrene sulfonyl chloride on the APTES-modified GF surface [112]. Luo et al. developed an ultraviolet graft copolymerization technique to graft acrylamide (AM) on the MGF surface. First, CH_2_· radicals were induced on the surface of MPTMS-modified GF under ultraviolet-lamp irradiation, which provided the active sites for grafting AM. AM was then polymerized on the GF surface to form AM-modified GF [63]. Aberem et al. successfully polycondensed nylon 6,6 on GF. The –OH groups on the GF surface first underwent an esterification reaction with adipyl chloride to form acyl-chloride-grafted GF, and then the amide reaction between the GF and diamino-1,6-hexane produced nylon-6,6-modified GF [2].

#### 2.2.2. Grafting Inorganic Nanomaterials

**Figure 15:**
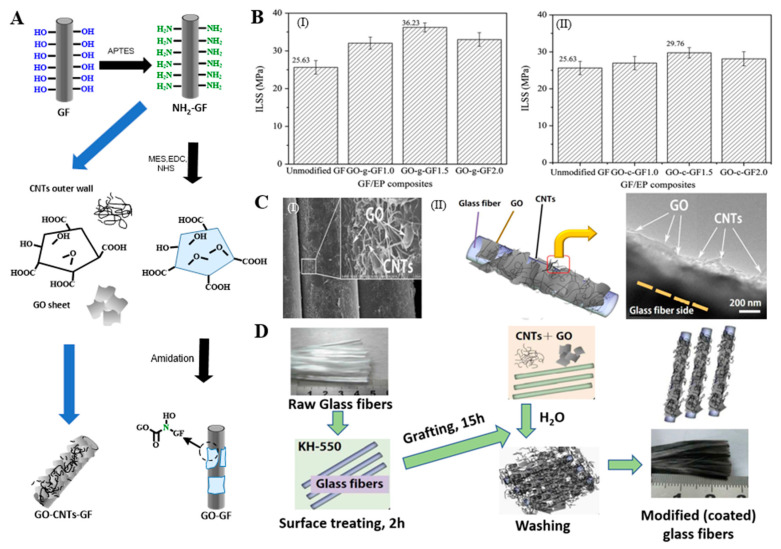
(**A**) The chemical routes to the synthesis of GO−g−GF are indicated by the black arrow. Schematic illustration of simultaneously grafting CNTs and GO onto one glass fiber, in which OH or HO represents the hydroxyl group, NH_2_ denotes the amino group, and COOH represents the carboxyl group. Silane-coupling agent for GF surface modification acts as a sort of intermediary that bonds organic materials to inorganic materials; this process is shown by the blue arrow. (**B**) Interlaminar shear strength of GF/EP composites. Reproduced with permission from reference [65]. Copyright 2014 *Composites Science and Technology*. (**C**) (**I**) SEM image (upper) and (**II**) HRTEM image (lower) of the GO/CNT hybrid coating layer on glass fiber surfaces. (**D**) Schematic showing grafting, coating, and washing processes for coating glass fibers. Reproduced with permission from reference [116]. Copyright 2017 *Composites Science and Technology*.

**Figure 16:**
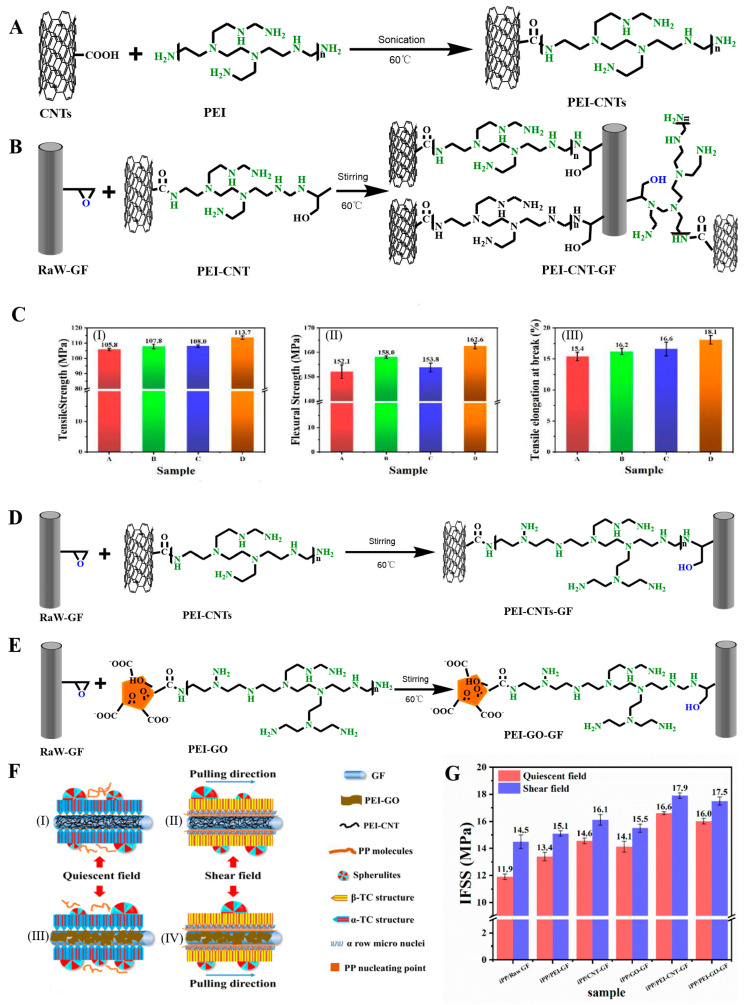
(**A**) Schematic diagram of PEI grafted to CNTs. (**B**) Schematic diagram of PEI−CNT reacted with GFs. (**C**) (I) Tensile strength, (II) flexural strength, and (III) tensile elongation at the break of different glass fibers reinforced with PA6 (A: PA6/Raw GF; B: PA6/PEI−GF; C: PA6/CNT−GF; and D: PA6/PEI−CNT−GF). Reproduced with permission from reference [119]. Copyright 2019 *Polymer*. Schematic representation of the graft reaction mechanism of (**D**) PEI−CNT on GF surface and (**E**) PEI−GO on GF surface. (**F**) Schematic diagram of mechanisms, under the quiescent condition: (**I**) iPP/PEI−CNT−GF and (**II**) iPP/PEI−GO−GF; under sheer field: (**III**) iPP/PEI−CNT−GF and (**IV**) iPP/PEI−GO−GF. (**G**) IFSS of various iPP/GFs micro−composites under quiescent and shear fields, respectively). Reproduced with permission from reference [120]. Copyright 2020 *Composites Science and Technology*.

Grafting of inorganic nanomaterials has mainly focused on graphene [65,114], CNTs [115], nanofibers [67], and nanoapatite (nHA) [23]. These inorganic nanomaterials have good dispersion, the preparation process is mature, and the nanomaterials have many active centers, and the chemical activity of the modified fiber surface is improved, which has become a hot spot for modified fibers. Chen et al. covalently grafted GO on the APTES-modified GF surface through an amidation reaction (Figure 15A). GO firmly adhered to the GF through amide bonds and changed the surface configuration of the GF [116]. Hua et al. used the same mechanism to graft mixed CNTs and GO on GF (Figure 15A,D) to prepare the GO/CNT-modified GF. Two-dimensional GO and one-dimensional CNTs were entangled and played the roles of encapsulation and anchoring, respectively, synergically enhancing the properties of the GF material (Figure 15C) [114]. Tzounis et al. chemically grafted multi-walled CNTs on the GF surface by the impregnation method (Figure 6C). APTES was blended with acyl-chloride-modified CNT (CoCl-CNT) solution, and covalent or noncovalent CoCl-CNT was grafted on the GF surface by amide bonds [70]. Tzounis et al. immersed GF in a carboxylated-CNT (COOH-CNT) solution, and COOH-CNT physically adsorbed on the GF surface through hydrogen bonding or electrostatic interaction [117,118]. Fang et al. grafted PEI-modified CNTs (PEI-CNTs) and GO (PEI-GO) on the surface of GF and successfully synthesized PEI-CNT-modified GF and PEI-GO-modified GF, respectively (Figure 16A,B). The PEI-CNTs and PEI-GO formed strong chemical bonds on the GF surface through the graft reaction, introduced abundant active functional groups, formed a dense and uniform network structure, and changed the surface morphological structure and chemical properties of the GF [119,120,121]. Sarr et al. impregnated GF in a cellulose nanofiber (CNF) suspension, and CNF was partially grafted on the GF surface through three-dimensional hydrogen bonding to the surface hydroxyl groups to form CNT-modified GF (CNT-GF) [67]. Siddiqui et al. grafted nHA on GF by microwave radiation, and nHA was uniformly dispersed on the GF surface in a spherical form [23].

### 2.3. Fluorination Modification

Fluorination of the GF surface is one of the most effective ways to produce FGFs. The fluorine atoms are contained in the SiO_2_ skeleton through covalent or ionic bonds. FGFs are an important and unique type of GF derivative. After optimizing the fluorination conditions, the prepared fluorinated materials have diverse properties, and the fluorine content is controllable. The FGF contains the Si–F bond type, and the characteristics are repeatable. Controlled reactions can be performed at room temperature with low energy consumption, and the reactions are green and fast with no human contact with toxic reactants. They can also neutralize F_2_ and the final fluorinated products remaining in the fluorinated reactor.

FGF is obtained using GF as the starting material with fluorine gas, xenon difluoride, or other organic/inorganic fluorine sources as the fluorination reagent. Different types of raw materials/fluorine sources and different reaction conditions are used to obtain FGFs with different degrees of fluorination. Fluorination modification methods can be divided into direct gas fluorination, solvent fluorination, plasma fluorination, and mechanochemical fluorination.

In the fluoridation process of GF, heteroatoms (e.g., argon, oxygen, hydrogen, or nitrogen) should be introduced into the fluoridation agent to ensure that the fluoridation degree of the GF is achieved in the shortest time. After sizing, the GF needs to be desized, or GF without sizing should be used before fluorination. GF without sizing can achieve perfluorination after sizing under mild fluorination conditions, but it may cause deflagration.

#### 2.3.1. Direct Gas Fluorination

Direct gas fluorination is a common method to prepare FGF using gaseous fluorination reagents, such as F_2_, HF, or other hybrid inert gases. By controlling the pressure, time, and temperature, the degree of fluorination can be easily adjusted, but the uniformity of fluorination needs to be further improved, and special equipment and strict safety procedures are required [122].

The fluorination reaction can be performed in the dynamic mode of an open reactor and the static mode of closed passivation of the reactor. In an open reactor, continuous introduction of the mixture can remove excess F_2_ and by-products. In a closed reactor, after the introduction of a specified amount of gas, excess F_2_ and by-products can be removed after the reaction by inert-gas flushing. The two methods are based on different reaction kinetics. In terms of the reactivity to molecular fluorine, the fluorination temperature is higher for a higher passivation degree of the GF. When the reaction gas is introduced at a high temperature with high reaction gas flow, attention should be paid to the risk of structural spalling of the GF [123].

Jung et al. prepared FGF by fluorination reaction of GF with a fluorine–argon mixture [124]. Sugiyama discussed the reactivity of GF to fluorine gas from the perspective of the fluorination temperature. They prepared FGFs with different fluorine contents using diluted fluorine gas in the temperature range of 298–473 K, and they found that the fluorine content of the GF is determined by the fluorination temperature [125].

#### 2.3.2. Solvent Fluorination

Solvent fluorination modification is also a feasible method to prepare FGF. FGF is synthesized with the participation of different liquid fluorine-containing sources (HF, fluorinated salt solution, and PTFE) [103]. Compared with direct gas fluorination, solvent fluorination has better uniformity owing to the uniform solvent environment. By changing the solvent type, the structure and properties of the FGF can be changed. In addition, the use of mixed solvents can synergistically regulate the structure and properties of the FGF, resulting in rich reaction chemistry. However, existing solvents are a threat to the natural environment, and the separation and purification processes of the solvents and FGF products are always complex and energy-consuming, resulting in high production costs.

**Figure 17:**
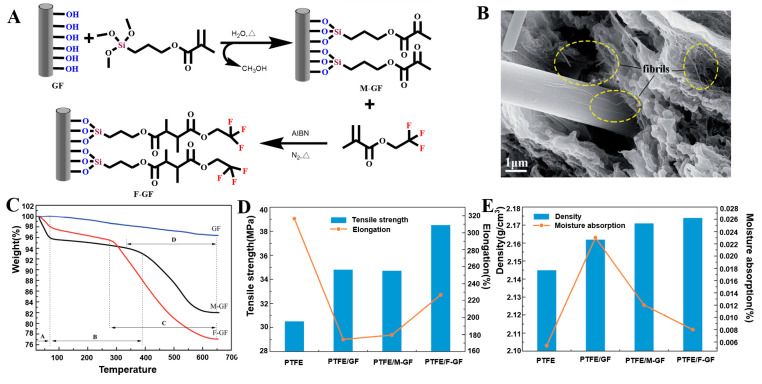
(**A**) The strategy for the surface fluorination of glass fiber. (**B**) The fibrils structure in PTFE/F-GF after a tensile fracture. (**C**) TG curves of GF, M−GF, and F−GF. (**A**) Residual solvent desorption; (**B**) physisorbed water desorption; and (C and D) modifier loss. (**D**) The tensile strength and elongation of PTFE, PTFE/GF, PTFE/MGF, and PTFE/F−GF. (**E**) The density and moisture absorption of PTFE, PTFE/GF, PTFE/M−GF, and PTFE/F−GF. Reproduced with permission from reference [127]. Copyright 2017 *RSC Advances*.

James et al. impregnated GF with three different fluoride concentration solutions (NH_4_F, NaF, and Na_2_SiF_6_). The fluoridation degree of the GF only depended on the concentration of the fluoride salt solution in the immersion bath. The optimal concentration of each salt was approximately 0.05 mol dm^−3^, which can add a small amount of fluorine to the GF surface without forming obvious salt deposits on the material [126]. Li et al. prepared FGF by blending γ-methylacryl-oxypropyl-trimethoxy-silane with 2,2,2-trifluoroethyl-methacrylate solution through a two-step process (Figure 17A). The hydroxyl groups on the GF surface reacted with γ-methylacryl-oxypropyl-trimethoxy-silane to generate a silane layer. The unsaturated double bond in the silane layer continued to polymerize with 2,2,2-trifluoroethylmethacrylate to generate FGF, and the fluorine-containing functional groups were successfully grafted on the GF surface (Figure 17B) [127]. Yin et al. produced a superhydrophobic coating on the GF surface. The GF was modified by mixing a synthetic high-fluorine-content acrylate emulsion and emulsifier. The GF surface was enriched with crosslinked fluorinated side chains. The hydrophobicity of the GF was significant, which is helpful for anti-fouling of the coating surface, and the method has potential applications in the manufacture of hydrophobic materials [128].

#### 2.3.3. Plasma-Assisted Fluorination

Plasma-assisted fluorination is another important method to prepare FGF. Compared with direct gas fluorination, plasma-assisted fluorination is cleaner and more controllable. In the plasma-assisted fluorination process, some of the ionized sources, such as SF_6_, CF_4_, and F_2_, are dissociated, and the formed fluorine free radicals adsorb on the GF surface, and heterofluoro groups are then generated in situ [122].

Daan et al. introduced a mixture of He/CF_4_ into a low-temperature plasma device to locally fluoridate GF of a specific size, and they investigated the influence of the plasma power on the fluoridation degree of the GF. X-ray photoelectron spectroscopy showed that fluorine was present both inside and outside the plasma-treated fiber bundle. In addition, the FGF showed higher fluorine content for higher plasma power. The spectrum also showed that an increase in the plasma power led to a larger peak value of fluorine [129,130].

#### 2.3.4. Mechanochemical Fluorination

Mechanochemical fluorination is a green method. With the aid of mechanical activation of the GF and fluoride ions, mechanical energy is converted into chemical energy. In the absence of a solvent, in the solid state, mechanical energy input by mixing, impact, and grinding produces instantaneous local high pressure and high temperature, achieving some unusual reactions. After mechanochemical fluorination, because the mechanical force decreases, the density increases, and the fluorine content is lower compared with that of other methods. Compared with direct gas fluorination, the reaction does not need any auxiliary solvent, does not require subsequent purification, and it beneficial for industrial production. However, it is difficult to track the reaction, and, as a result, the corresponding mechanochemical mechanism is unclear [122].

### 2.4. Other Modification Methods

#### 2.4.1. Redox Modification

Oxidation modification of GF refers to using an oxidant to oxidize the functional groups on the GF surface at an appropriate temperature to increase the content of oxygen-containing functional groups on the surface. Commonly used oxidants include HNO_3_, HClO, H_2_O_2_ solution, and O_3_. Reduction modification of GF refers to a reduction of the functional groups on the GF surface at an appropriate temperature, and the chemical properties of the surface are changed. Commonly used reducing agents include He and H_2_.

Guo et al. oxidized GF cotton with two inorganic reagents: hydrogen peroxide and piranha solution (98% concentrated sulfuric acid and 30% hydrogen peroxide solution mixed at a volume ratio of 7:3). SEM was performed to characterize the surface morphology of the GF before and after modification. The non-oxidized MGF (FG-0) had a smooth surface. The sample modified by hydrogen peroxide oxidation (FG-1) showed a small number of pore structures of different sizes on the surface. The GF treated with piranha solution (FG-3) showed obvious etching traces and an uneven surface [22].

#### 2.4.2. Acid–Base Etching Modification

Acid–base etching modification forms some depressions or microholes on the GF surface by soaking the GF in acid–based solution [131,132], thus significantly increasing the surface area of the GF and the number of active functional groups on the surface. Acid treatment changes the GF surface through an ion-exchange reaction, and H^+^ in the acid replaces other metal cation components, such as Ca^2+^ and Al^3+^. During alkali treatment, the alkali directly attacks the silica network. The Si–O–Si bond is broken by an attack of OH^−^. Alkali treatment can affect GF more than acid treatment. The treatment effect of this method is mainly related to the acid and base types, solution properties, concentration, temperature, and time [34]. Commonly used acid modifiers are hypochlorous acid, phosphoric acid, hydrochloric acid, and citric acid. Alkaline modifiers include sodium hydroxide, ammonia, and potassium hydroxide. In addition, acid–based etching can significantly enhance the tensile strength of the GF by removing surface defects, changing the damaged surface, and decreasing the fiber diameter.

Aminian et al. soaked GF in 90 °C hydrochloric acid solution for 2 h and compared the X-ray photoelectron spectra before and after hydrochloric acid soaking. After hydrochloric acid treatment of the GF, the sodium peak disappeared, the carbon peak intensity decreased, and the silicon peak and oxygen peak intensities were enhanced, becoming sharper and more intense. The hydrochloric acid solution can remove impurities adsorbed on the GF surface and expose silicon and oxygen atoms on the GF surface [82]. Guo et al. modified GF cotton with a sodium hydroxide solution. Compared with the smooth surface of the fiber before modification, the GF surface after alkali modification showed membrane-like fibers accompanied by a large amount of fiber agglutination, which increased the surface area of the GF and the number of active functional groups on the surface. This was closely related to strong corrosion by the sodium hydroxide solution.

#### 2.4.3. Plasma Treatment Modification

The principle of plasma processing technology is as follows: in the reactor, the electrons forming the plasma obtain sufficient energy to initiate dissociation, ionization, or attachment reaction with molecular free radicals or atoms, which is a very effective reaction for silicon compounds.

Plasma treatment is used for physical and chemical modification of the GF surface to improve its surface properties and for modification of the interfacial properties between the multiple components of fiber composites [133]. Plasma technology has two functions: etching, including surface cleaning (removal of organic pollutants) and ablation (elimination of weak areas and increasing the surface roughness), and fiber surface functionalization, depending on the materials and conditions. However, the plasma polymer shows easy sedimentation in the plasma chamber, limiting the industrialization of plasma treatment for GF modification [134,135].

Plasma treatment can be used to deposit protective, barrier, biocompatible, or multifunctional films with customized physical, chemical, and surface properties on fiber surfaces with controlled adhesion. Depending on the number and function of the reactor segments, the films can be deposited as monolayers, multilayers, or gradient nanostructures [136].

Cech et al. used a radio-frequency helical coupled plasma system to continuously modify the surface of GF (Figure 4B). The plasma polymers hexamethyldisiloxane (PP-HMDSO) and VTES (PP-VTES) in thin film form were deposited on the GF surface. The PP-HMDSO and PP-VTES MGFs were highly crosslinked, and with increasing power, the degree of crosslinking of the continuous random carbon, oxygen, and silicon network increased [137]. The surface modification process of GF was optimized by Cech and his group using roll-to-roll plasma equipment. A plasma polymer film of tetravinyl silane was deposited on GF pretreated with oxygen-argon plasma. By changing the deposition parameters, the consumption and deposition rates of the polymer film were controlled, and then the thickness and properties of the polymer film could be controlled [37,38,136].

#### 2.4.4. Doping Modification

GF can be chemically modified by doping to enhance its physical and chemical properties. The main doping methods are the element-doping method, oxide-doping method, and carbon-material-doping method. Xiao et al. prepared black phosphorus (BP)/polyethylene terephthalate (PET)-modified GF and red phosphorus (RP)/PET-modified GF by adding BP and RP, respectively, to a mixture of GF and PET [40]. Liverani prepared electrospun copper-doped GF by a combination of electrospinning and sol–gel techniques. The results showed that copper was successfully incorporated into the GF [39].

## 3. Characteristics of MGF

The characteristics of MGF depend on the MGF itself (volume, length, and orientation angle) [138,139], modification, modification method [140], and subsequent working conditions [141,142].

MGF modification technology determines the characteristics of the functional groups attached to the GF, including the contents, locations, and types of functional groups, and it greatly changes the surface structure of the GF. The modification method, raw materials, and modification conditions also significantly affect the structure, properties, and corresponding application fields of MGF, resulting in unique physical and chemical changes.

### 3.1. Surface Structure

To understand how modification methods affect the surface structure of GF, X-ray diffraction, Raman spectroscopy, and SEM have been performed. These analyses show the influence of the modification method on the fiber diameter, specific surface area, and coating thickness.

#### 3.1.1. Diameter Evolution

The fiber diameter generally decreases slightly with modification, which is attributed to partial etching of the fiber and removal of structural defects. Compared with the silicon-fiber region, the amorphous part of the surface is more reactive to modification, and it is more conducive to etching. However, an increase in the fiber diameter has also been observed, which is attributed to coating, functional group coverage, and grafting on the surface of the fiber. When both mechanisms occur, the effect of the increase in the diameter is more prominent than that of the decrease in the diameter [123].

Yang et al. reported that HF etching caused the relative diameter of GF to decrease to 15% of that of the unmodified GF [13]. Bashir used NaOH and KOH to study the effects of the temperature, concentration of the alkaline solution, and treatment time on the reduction of the GF diameter. In the same alkaline solution, the reduction of the GF diameter linearly increased with time, and the reduction rate of the diameter was also linearly correlated with the concentration of the KOH or NaOH solution. The rate increased with increasing concentration, and the diameter reduction rate and temperature increase followed an exponential model. The rate increased with increasing temperature, and the corrosion degree of GF treated with NaOH was twice that of the GF treated with KOH [35]. By covering the GF surface with iron ore nanoparticles, Gutierrez et al. greatly changed the GF surface morphology. There were massive protrusions on the GF surface, the specific surface area was comparatively larger, and the fiber diameter was larger (7 mm larger than that of the unmodified GF) [84]. Fang et al. investigated the relationship between the diameter of RGO-coated GF and the number of dip-coating cycles. The diameter linearly increased with the increasing number of dip-coating cycles, from 22.8 μm for the undipped fiber to 42.5 μm for the fiber dip coated 20 times. Therefore, the thickness and quality of the graphene coating can be effectively controlled by adjusting the number of dip-coating cycles [87].

#### 3.1.2. Surface Area

The surface structure of a fiber material determines the properties of the fiber. Modification can smooth the GF surface by weakening the fiber stripes and covering it with a uniform coating, reducing the specific surface area. In addition, the fiber surface can become rough and damaged by removing the weakly bonded oxy–silicon surface layer, and the fiber surface shows more obvious stripes and holes. The uneven distribution of various functional group coatings grafted on the GF surface will lead to an increase in the specific surface area. When both roughing and smoothing simultaneously occur, roughing precedes smoothing, and the relative specific surface area increases [123].

Thomason reported that by modifying the GF surface with a silane coupling agent, a crosslinking layer formed on the GF surface, which appeared as “islands” instead of a continuous layer on the GF surface, resulting in an uneven structure (Figure 3C). Thus, the specific surface area increased [64,65,143]. Tomao soaked GF in 20% hydrochloric acid solution and studied the influence of the treatment time (4, 6, and 8 h) and temperature (40, 60, 80, and 100 °C) on the surface hydroxyl density on the GF. The surface hydroxyl density of the MGF was higher for higher treatment temperature and time. When the GF was soaked at 100 °C for 6 h, the number of hydroxyl groups on the MGF surface was the largest, with minimal changes in the fiber structure. Tomao analyzed the adsorption isotherm of gas and H_2_O by the Brunauer–Emmett–Teller equation, and they measured the surface area and pore-size distribution of the GF before and after HCl hydrothermal treatment. The contactable surface area of the GF significantly increased after treatment, expanding to 70 times that of the unmodified GF. Moreover, according to the N_2_-adsorption isotherm and Horvath–Kawazoe model, the pore type was determined to be micropores of approximately 0.6 nm in diameter, which explains the high surface area of the MGF [36].

#### 3.1.3. Coating Thickness

The coating thickness can be estimated by treating the fiber as a two-phase material, with the outer coating and GF body as the two phases. The coating thickness depends on the modification method, raw material, and modification conditions. By changing the modification conditions and increasing the treatment duration, temperature, and pressure, the coating thickness continues to increase, meaning that the reaction is more thorough the longer the coating reacts with the GF [123].

Cheng et al. reported that the treatment time could determine the number of graphene layers formed on GF. One to two graphene-film layers formed when the treatment time was 10 min, and the number of graphene-film layers reached 3 to 5 when the treatment time was 20 min [50]. Gao investigated the effect of the pyrolysis temperature on the thickness of the graphene coating on the GF surface. In a certain range, the coating thickness nonlinearly increased with increasing temperature. In the temperature range below 700 °C, there was no obvious coating on the GF surface. The thickness of the graphene coating at 750 °C was 6 nm, and it did not exceed 20 nm below 800 °C. When the pyrolysis temperature was 900 °C, the coating thickness increased to approximately 46 nm. When the pyrolysis temperature exceeded 900 °C, the coating thickness continued to increase, but the damage to the GF matrix at high temperatures also increased, and heating enhanced the pyrolysis process and coating growth [51]. Tamrakar et al. controlled the thickness and uniformity of a PEI-CNT coating deposited on the GF surface by adjusting the processing parameters during electrophoretic deposition, including the electric-field intensity, deposition time, and CNT-dispersion concentration. By controlling the electric-field intensity to 15 V/cm and adjusting the deposition time from 7.5 to 10.5 to 15 min, the PEI-CNT film thickness increased from 200 to 400 to 600 nm, the CNT-film thickness doubled, and the film uniformity improved. Compared with adjusting other parameters, adjusting the deposition time was considered to be the most effective method to adjust the thickness of the PEI-CNT coating [95]. Sarr et al. evaluated the evolution of the CNT-GF surface structure by the CNF graft rate, CNF-layer thickness, CNF-cluster density, and average cluster length. When the concentration of the CNT impregnation solution was increased from 0.001 to 0.5 wt%, all four properties increased to varying degrees, and a higher concentration of the impregnation solution caused higher aggregation rate. With increasing concentration of the CNT impregnation solution from 0.05 to 0.5 wt%, the grafting rate increased from 71.7% to 72.4%, the thickness increased from 279 to 1248 nm with a growth rate of more than 300%, the CNF-cluster density increased from 0.688 to 0.848, and the average cluster length also increased [67].

### 3.2. Wettability

Wettability is a key factor that determines the processing and use of polymer materials. Changes in the surface morphology and chemical properties affect the wettability and surface energy of GF. Higher surface energy results in higher wettability and improves interfacial adhesion between the GF and matrix. It is also commonly used as an indicator of the surface modification (chemical or physical) nature or degree of polymer materials. The equilibrium contact angle between a liquid and a solid surface is commonly used as a measure of the wettability of the liquid on the solid. Changes in surface energy and wettability can be used to customize MGF as a polymer or as a reinforcement material in a polymer [57,119].

Unmodified GF has good hydrophilicity. It can aggregate by hydrogen bonding and react with water, limiting the application range of GF [144]. To improve the hygroscopicity of GF, GF is modified to enhance the hydrophobicity or hydrophilicity, depending on the modification technology used and the surface structure formed.

Increasing the hydrophilicity of MGF increases the wettability with several liquids, such as water, glycerin, or fluoropolymers. This enhanced wettability is related to a sharp increase in the polar components of the surface energy (e.g., formation of –OH), resulting in an increase in the total surface energy.

The hydrophobicity of GF can be increased by forming hydrophobic functional groups, depositing a hydrophobic coating, and fluorination. Increasing the hydrophobicity decreases the wettability, increases the contact angle, and decreases the surface energy. The hydrophobicity can be increased in two ways: silicon-based functional groups, nonmetallic coatings, and fluorination [128] can directly increase the GF hydrophobicity, whereas silicon-based nonpolar functional groups, the carbon–carbon bonds of nonmetallic coatings, and the high carbon–fluorine ratio of fluorinated coatings increase the hydrophobicity by resulting in fewer hydrophilic ions exposed on the GF surface. Although the treatment initially increases the hydrophilicity of the GF, when the parameters are varied, such as the temperature, coating thickness, degree of fluoridation, treatment duration, and pressure, or gas mixture composition, the hydrophobic contribution can be greater than the hydrophilic contribution [123].

Thomason reported that different silanes could significantly change the wettability of the GF surface. With increasing silane content on the fiber surface, the fiber surface polarity gradually decreased, the adhesion of water decreased, and the wettability decreased [60]. Fang reported RGO-coated GF with high hydrophobicity. The contact angle gradually increased with an increasing number of dip-coating cycles. The contact angle increased from 23° for the GF without dip coating to 105° for the GF after five dip-coating cycles. After five dip-coating cycles, the contact angle remained stable with a further increasing number of dip-coating cycles. This was attributed to the inherent hydrophobicity of the graphene coating and the roughness of the coated GF, and the thickness of the coating increased with increasing number of dip-coating cycles [87]. Li et al. prepared FGF and compared it with the unmodified GF. The FGF showed good dispersion and lower aggregation than the unmodified GF, which was attributed to the presence of CF_3_ enhancing the hydrophobicity of the FGF [127]. James et al. conducted a series of single-fiber contact-angle measurements in different fluoride concentration solutions (NH_4_F, NaF, and Na_2_SiF_6_). All of the fluorinated fibers showed higher hydrophobicity than the untreated GF, which was related to the degree of fluorination of the GF surface and the inherent properties of the fluoride solution [126]. Daan et al. conducted dynamic micro-wetting experiments. The average wetting rate of FGF cotton treated by He/CF_4_ with different plasma powers was significantly lower than that of the untreated GF, mainly because the introduction of fluorine atoms increased the hydrophobicity of the GF [129,130]. Siyal et al. evaluated the hydrophobicity of FG-modified GF film (FG-GF) under different pH conditions and for different FG particle numbers. The FG coating could withstand pH values of 3–11 and lost its hydrophobicity at pH values of 1 and 13. FG increased the hydrophobicity and particle size, which was attributed to the C–F group in the FG particles. Siyal et al. evaluated the amphiphilicity of FG-PDMS-modified GF (FG-PDMS-GF) film. The FG-PDMS-GF film showed stronger hydrophobicity than the significant hydrophobicity of the FG-GF and enhanced hydrophobicity of the PDMS-modified GF [92]. Hamid et al. coated the surface of GF with PDMAA, PS, and PFA polymers with different surface energies to adjust the wetting behavior of GF (Figure 2C). The GF surface changed from super hydrophilic to very hydrophobic [61]. Luo et al. compared water absorption in AM-modified GF in different treatment stages. The water-absorption rate in the AM-modified GF increased from 13.6% to 23%, which was related to the presence of hydrophilic polyacrylamide [63].

### 3.3. Electrical Performance

Changing GF from an insulator to a conductor or a semiconductor causes significant changes in the overall electrical properties of the GF. The electrical properties, including the resistance, resistivity [48], and conductivity [123], change as the coating type, homogeneous degree, and degree of interconnection of the conductive path change. The conductivity of metallic-coating GF is higher than the conductivity of nonmetal-coated GF. The conductivity of a uniform and dense coating is higher than that of an uneven, loose coating, and the conductivity of a conductive path with high interconnection is higher than that of a conductive path with low interconnection [87].

Lien et al. compared the mechanical and electrical properties of electro-less silver-plated GF with different TEOS-concentration pretreatments. According to the results of ball-milling experiments, the TEOS concentration was proportional to the amount of silver plating, and an appropriate TEOS concentration improved the adhesion between the GF and silver-plated layers. When the TEOS concentration was 5 or 10 g/L, the enhancement effect was the best, showing the lowest resistance and best mechanical stability [44]. Xu et al. improved the conductivity of copper-plated GF by optimizing the pretreatment process of the copper-plated GF, the formula of the plating solution, and the conditions of electroplating. The volume resistivity was as low as (10^−5^ Ω·cm^−1^) [45,48], which was better than those of silver-plated GF (10^−4^ Ω·cm^−1^) [46] and nickel-plated GF (10^−3^ Ω·cm^−1^) [77]. Tamrakar et al. found that the resistance of PEI-CNT-modified GF decreased with increasing electric-field intensity and deposition time (Figure 8C), which was related to the increase of the PEI-CNT conductive film thickness and enhancement of the uniformity [95]. Tzounis et al. studied the conductivity of CNT-GF (Figure 6D,E). The average conductivity of the CoCl-CNT-modified GF (20 S/m) was 10 times higher than that of the COOH-CNT-modified GF (2 S/m), and the average resistance of the CoCl-CNT-modified GF (10^4^–10^5^ Ω) was approximately one-tenth of that of the COOH-CNT-modified GF (10^5^–10^6^ Ω). This was attributed to the highly entangled and tightly packed network distribution and more uniform microstructure of the CoCl-CNTs than the COOH-CNTs [70]. Fang et al. reported graphene-coated GF with high conductivity. With an increasing number of dip-coating cycles, the coating resistance sharply decreased with the dip-coating time, and the volume conductivity sharply increased, reaching a maximum of 24.9 S/cm. This was attributed to the high conductivity of GO, the full-coverage structure of the GF, and the efficient electronic transmission network. Furthermore, the conductivity was strongly dependent on the dip-coating time. For a longer dip-coating time, the graphene coating was thicker, there were more conductive paths, and the resistance was lower [87]. Gao et al. measured the conductivity of graphene-coated GF prepared at different pyrolysis temperatures. They found that the resistance of the coating decreased with increasing temperature, while the conductivity showed the opposite trend. The resistance values of the coatings prepared at 800, 850, and 900 °C were 1.9 × 10^6^, 4.6 × 10^5^, and 2.4 × 10^5^ Ω, and the conductivities were 2.87 × 10^5^, 4.5 × 10^5^, and 8.1 × 10^5^ S/m, respectively. Better conductivity was obtained for a smaller coating thickness [51].

The conductivity of graphene-coated GF was compared with the conductivities of other GF coatings reported in the literature. The conductivity of RGO-coated GF prepared by dip-coating technology was 24.9 S/cm, which was higher than that of the CNT-coated GF of 20 S/cm and polyaniline-coated GF (6 S/cm), and the electrical conductivity of graphene-coated GF prepared at a pyrolysis temperature of 900 °C was 0.5 S/cm. The conductivity of RGO-coated GF was also six orders of magnitude higher than that of GF modified with an NH_2_-based graphene-nanosheet coating ((5 ± 1) × 10^−4^ S/cm) and nine orders of magnitude higher than that of GF modified with an NH_2_-based graphene-nanosheet coating ((9 ± 7) × 10^−7^ S/m) [93].

### 3.4. Mechanical Properties

Modification can maintain or even improve the mechanical properties of GF [145,146], paving the way for the interaction between MGF and polymers in composite materials and polymers. The evolution of the mechanical properties (Young’s modulus and tensile strength) of MGF under specific modification conditions has been investigated.

The ultimate tensile strength and Young’s modulus of GF sharply increase with modification. The improvement of the mechanical properties can be explained by the elimination of surface defects and the strengthening of the interaction between the modified functional groups and the GF surface. Drastic modification technology leads to surface damage and the formation of pits, and the mechanical properties significantly decrease. Other properties, such as adhesion or hydrophobicity, can be obtained at the expense of mechanical properties. Reduction of the mechanical properties does not necessarily result in the MGF not being a reinforced composite material [123].

Thomason reported that the single-fiber tensile strength of GF depends on the chemical structure of the selected silane, with the bonding to the fiber surface and the formation of siloxane networks increasing with the increasing number of alkoxy groups attached to the silicon atoms. This protects the GF from the most serious surface defects and improves the average fiber strength. Compared with the single-fiber tensile strength of the original unsized GF pulp, the single-fiber tensile strength of the GF cotton significantly increased. The sizes of the two main ingredients, the silane-coupling agent and film-forming polymer, synergistically affect the fiber strength. The silane-coupling agent provides water protection and/or eliminates the weakest filament fibers. The film-forming agent reduces the generation of new defects and promotes the healing of old defects. In addition, the defect repair hypothesis explains the improvement of the fiber tensile strength, and sizing affects the number and size of the fiber surface defects through the repair mechanism [61]. APTES pulp fiber has the highest average strength, and it is higher than the average strength of only silane-sizing fibers. The single-fiber tensile strength of silane-treated GF is higher than that of the water-based slurry. After elastomer coating, the fiber tensile strength is further enhanced. Moriche et al. tested Young’s modulus and mechanical strength of GNP-modified GF and NH_2_-GNP-modified GF by nanoindentation. Compared with those of the unmodified GF, Young’s modulus values of the GNP-modified GF and NH_2_-GNP-modified GF increased by 31% (21 ± 2 GPa) and 162% (42 ± 2 GPa), and the hardness increased by 50% (3 ± 1 GPa) and 350% (9 ± 1 GPa), respectively, owing to the strong interaction between NH_2_-GNP and GF [93]. Yang et al. studied the effect of HF treatment on the tensile strength of heat-treated GF. They found that short-term treatment in low-concentration HF solution enhanced the tensile strength of the GF. For GF treated at different temperatures (450–600 °C), soaking in dilute hydrofluoric acid (HF) for 0.5 min increased the tensile strength of the GF by two times, and the tensile strength increased by three times after 2.5 min. HF improves the tensile strength of GF by removing surface defects and reducing the fiber diameter [13].

### 3.5. Stability

Owing to the special network structure, GF has good thermal and chemical stability, but the stability of MGF is different from that of GF. In MGF, different types of functional groups, the binding abilities of the functional groups, and the degree of defects will affect the thermal and chemical stability of the MGF. On the one hand, MGF is coated with a chemically stable coating to protect the GF from damage caused by temperature and mechanical shock, which improves the GF stability. On the other hand, the surface active functional groups of the MGF are easily removed and damaged under high temperature and pressure, resulting in lower stability of the MGF than the GF.

Xu et al. used the thermal shock method to investigate the stability of the copper coating on the surface of copper-coated GF. There was no peeling, bubbling, or shedding on the surface of the coating after the thermal shock, which showed that there was a strong interaction between the coating and GF. In addition, the oxidation stability of the copper-coated GF was confirmed by monitoring the volume resistivity of the copper-coated GF for different days of exposure [48]. Li et al. analyzed the thermogravimetric curves of GF, MGF, and FGF composite materials (Figure 17C). The failure of the MGF and FGF composite materials was divided into three temperature regions. The first region corresponded to the analytical temperature of the solvent, the second region corresponded to the analytical temperature of the physical adsorption of water, and the third region was considered to be the complete oxidation of the material. However, the MGF and FGF were only completely oxidized and destroyed near the third region. The two composite materials showed good heat resistance, but their thermal stability was lower than that of the GF [127].

## 4. Applications of MGF

**Figure 18:**
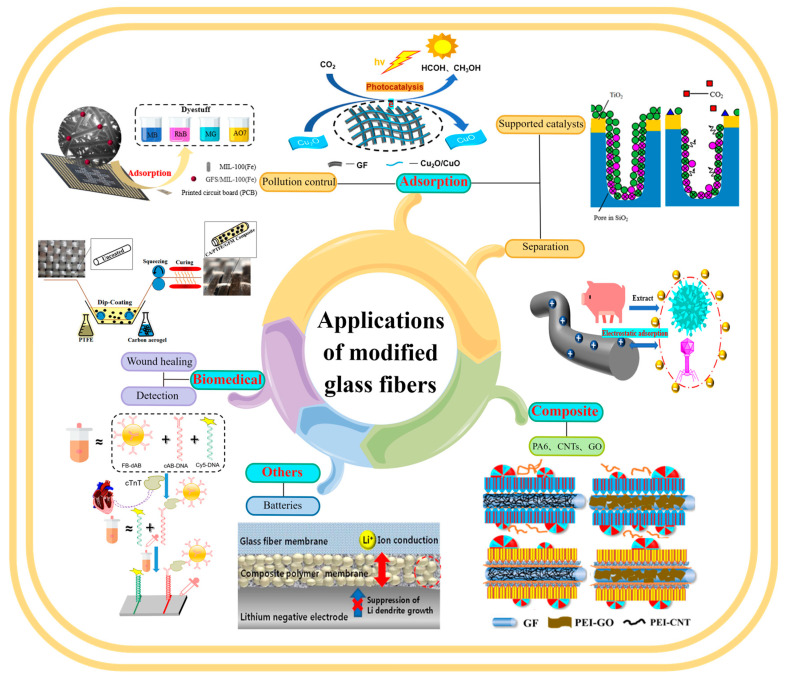
Overview of the application of modified glass fibers diagram (by Figdraw).

GF is used in a wide range of fields, as described in Figure 18. It is an important material in the fields of adsorption, reinforced polymers, and biomedicine and is widely used in environmental protection, petrochemicals, building materials, aviation and national defense, biomedicine, and other fields [142,147].

### 4.1. Adsorption

MGF has uniform micropores, high micropore volume, and high surface area, and it shows excellent adsorption performance. In addition, the chemical groups on the GF surface, surface chemical properties represented by the hydrophilicity/hydrophobicity, and charge on the GF surface make the adsorption characteristics of GF very effective in adsorption fields [82], such as material separation [18], pollution control, multifunctional carriers, and gas storage.

#### 4.1.1. Separation

MGF shows high adsorption capacity and fast binding ability with target molecules. It can effectively analyze and separate small inorganic molecules (e.g., CO_2_), organic pollutants (e.g., dyes), and biological macromolecules (e.g., proteins) [105].

**Figure 19:**
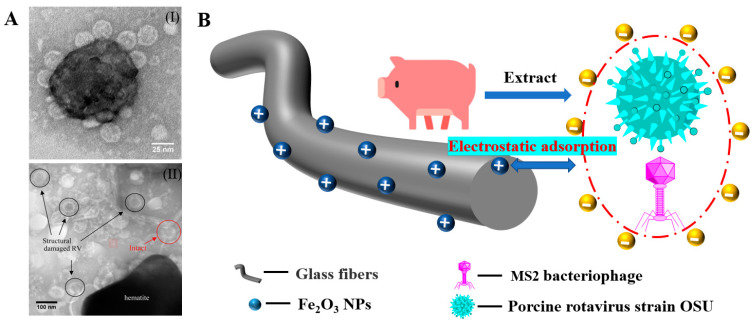
(**A**) (**I**) TEM image shows MS2 phages adsorbed to the surface of an aggregate of hematite nanoparticles. There was no noticeable decrease in the diameter of the viral particles, and their integrity did not appear to be compromised. (**II**) TEM image shows RV particles with compromised structure after coming into contact with hematite nanoparticles in solution. Reproduced with permission from reference [147]. Copyright 2009 *Water Research*. (**B**) Schematic diagram of hematite-coated fiber adsorption of rotavirus and phage by electrostatic action.

Sugiyama reported selective and enhanced adsorption of CO_2_ on FGF at 298 K. This adsorption behavior makes reactive GF a promising adsorbent for the separation and capture of CO_2_ gas in industrial streams [125]. Zhao et al. synthesized AuNP/MoS_2_/GF composites and then used them for quantitative detection and separation of trace crystal violet (CV) and toluidine blue (TB) (Figure 9B(I)). CV and TB were successfully separated by AuNP/MoS_2_/GF based on the different polarities of CV and TB [71]. Cao et al. used a super hydrophilic GF–G–PEI film for oil/water (O/W) separation. The abundant hydrophilic amine groups on the surface of HPEI-modified GF can combine with water molecules through hydrogen bonding (Figure 14C). In different emulsified and dispersed O/W mixtures, the quantitative oil removal rate was as high as ≥99.6%. The O/W dispersion performance of the HPEI-modified GF membrane was excellent, showing its promise for the development of a new O/W filter with high permeability, high repulsion, and durability [109]. Siyal et al. used an FG-GF membrane for direct-contact membrane distillation. The desalting effect was the best for FG-GF with a mass of 0.1 g, and the film flux remained stable for 300 min. The desalting rate remained above 97.5% after 840 min, indicating the excellent performance of the FG- (0.1 g) coated film in direct-contact membrane distillation [92]. Chen et al. used short-chain silane-derivative-modified GF (Figure 10A) for biological macromolecule separation (separation of lysozyme and albumin). The experimental results showed that the adsorption of long-carbon-chain-crosslinked or triethoxy silane was comparatively more hydrophobic and the protein adsorption ability was stronger than short-carbon-chain or unmodified GF, and the lysozyme adsorption capacity was higher than that of albumin. Subsequently, according to the hydrophobic interaction and desorption conditions of the GFM, lysozyme and ovalbumin were effectively separated in the two-step elution process, wherein 98% of lysozyme was recovered from 70% acetonitrile and 75% of convalbumin was recovered from 70% isopropyl alcohol [104]. Gutierrez et al. used GF coated with hematite nanoparticles to remove rotavirus (RV) and bacteriophage (MS_2_). MS_2_ and RV adsorbed to and were removed by the GF through electrostatic interaction (Figure 19). RV was inactivated owing to structural damage in the adsorption process, which resulted in irreversible adsorption. In the process of disinfection of drinking water, a filter element made of an iron-oxide-coated GF filter showed very strong application prospects [84]. Su and co-workers used a C_18_ hydrophobic membrane (Figure 10B) for adsorption separation of the crude extract from ginkgo biloba leaves in terpene lactones (the terpene lactone components usually include ginkgo lactone (BB), ginkgo lactone A, and ginkgo lactone B). The results showed that the modified C_18_ membrane showed better adsorption performance than commercial C_18_ solid-phase extraction adsorbents. The modified C_18_ film showed better selective adsorption performance for BB, and different desorption solvents were tested. The terpene lactones in the modified C_18_ film, especially BB, showed different adsorption and desorption characteristics for different components of ginkgolides according to the modified C_18_ film. Selection of an appropriate elution gradient achieved separation and concentration of the different terpene lactone components [105].

#### 4.1.2. Pollution Control

MGF shows high activity for the adsorption of organic matter and inorganic pollutants, including organic dyes, volatile organic compounds, and heavy-metal ions. However, there is no effective way to enrich the pollutants produced by MGF during regeneration.

**Figure 20:**
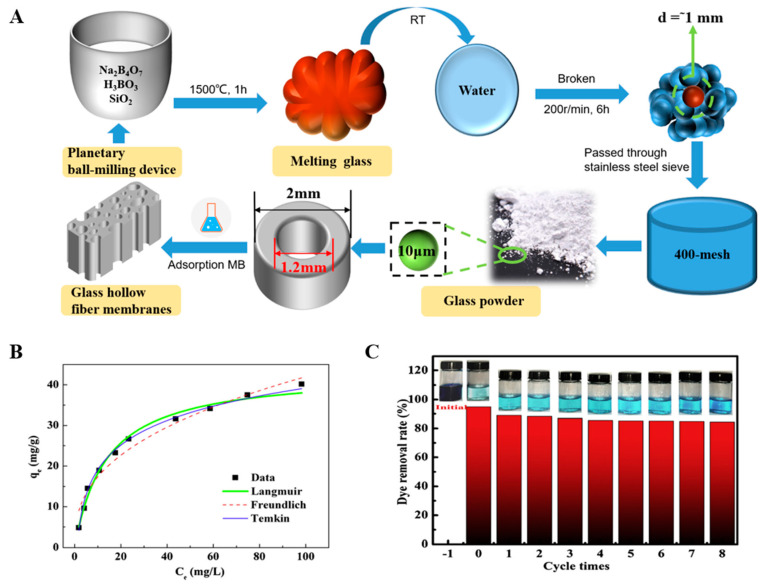
(**A**) Preparation of glass hollow-fiber membranes. (**B**) Adsorption isotherms and their simulations by three isotherm models for as-prepared membrane. (**C**) The removal ratio of the as-prepared membrane and the glass membrane after eight adsorption-calcination cycles. Reproduced with permission from reference [148]. Copyright 2019 *Journal of the European Ceramic Society*.

**Figure 21:**
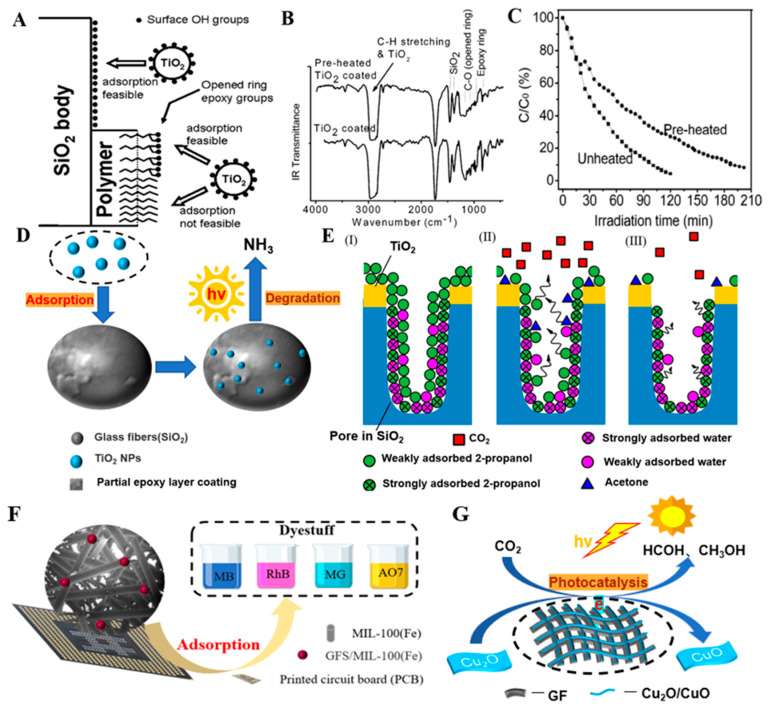
(**A**) Illustration of interactions between a TiO_2_ nanoparticle in solution and the surface of SiO_2_, the opened ring epoxy groups, and the organic chains of the layer on the surface. (**B**) FT−IR spectra of the coated glass fibers with and without preheating at 500 °C. It can be seen that the peaks of the epoxy ring and C−O (due to opened rings) are reduced after heating. (**C**) Photocatalytic degradation of ammonia for preheated and unheated glass fibers coated with TiO_2_ nanoparticles. Reproduced with permission from reference [82]. Copyright 2007 *The Journal of Physical Chemistry C*. (**D**) Adsorption and photocatalytic decomposition of gaseous 2−propanol using TiO_2_−coated porous glass fiber cloth. (**E**) Schematics of (**I**) adsorption, (**II**) early-phase photocatalytic oxidation, and (**III**) late−phase photocatalytic oxidation on the TiO_2_−coated porous glass cloth. Reproduced with permission from reference [81]. Copyright 2019 *Catalysts*. (**F**) Schematic diagram of synthetic glass fiber ball adsorbing dye in water. (**G**) The CuO coating on the glass fiber photocatalytic reduction of CO_2_ to HCOH and CH_3_OH.

Li et al. used an APTMS–GF composite membrane to selectively remove and separate the dyes Congo red (CR), bromophenol blue (BPB), methylene blue (MB), and methyl orange (MO) from binary solutions (Figure 12D). Based on electrostatic interaction, the APTPS–GF composite membrane (–NH^2+^) selectively removed CR (anionic dye) from mixed solutions of BPB/CR, MB/CR, and MO/CR, separating BPB (neutral dye), MB (cationic dye), and MO (weak anionic dye), respectively (Figure 12C) [64]. Zhang et al. prepared a glass hollow-fiber membrane with an asymmetric nanopore structure by phase conversion, sintering, and acid leaching, which was used for the removal of MB dye (Figure 20A). The influence of the initial adsorption rate was up to 95%. On the basis of the adsorption kinetics and thermodynamic data fitting, the highest degree was for the secondary model and Temkin model fitting, showing a chemical adsorption process and a spontaneous endothermic adsorption process (Figure 19 and Figure 20). After eight adsorption/calcination cycles, the removal rate of MB was approximately 85% (Figure 20C) [148,149]. Guan et al. used a GF ball (GFS) as a MIL-100 (Fe) carrier to synthesize the GFS/MIL-100 (Fe) composite (Figure 21F). Mil-100 (Fe) showed good dispersion in the synthesized GFS/MIL-100 (Fe) composite, and the load rate was up to 27.5%. The GFS/MIL-100 (Fe) composite was used to treat dye wastewater and showed good RHB removal performance. It also had a certain removal effect on MB, acid orange 7, and malachite green, and it can be reused, showing the potential of the GFS/MIL-100 (Fe) composite for the treatment of dye wastewater [150]. Guo et al. reported that MGF is a promising adsorbent for volatile benzene series compounds (VBSCs). They used 10 typical VBSCs (phenol, benzoyl chloride, aniline, benzene, xylene, benzyl alcohol, salicylic aldehyde, toluene, benzaldehyde, and styrene). The adsorption capacities of the VBSCs on the unactivated, activated, and aryl-modified GF were evaluated (Figure 11B). The adsorption efficiencies of the VBSCs on the activated GF were higher than those on the unactivated GF. The adsorption efficiencies of aniline, salicylic aldehyde, benzyl alcohol, and xylene on the aryl-modified GF were significantly enhanced, with the adsorption efficiency of benzyl alcohol being as high as 93%. The high adsorption efficiency is related to hydrogen bonding and π–π conjugation interaction (Figure 11C). The experimental results were consistent with SEM results, in which the activated and aryl-modified GF showed larger surface areas and more porous structures than the raw materials [22]. Letenkova studied the adsorption capacities of Cd(II) on three different GFs (BSTV basalt fiber, M20-STV-2.0, and M20-UTV-0.6 GF). All three GF materials showed high adsorption capacity for Cd(II). Based on the distribution coefficient and adsorption equilibrium constant of the Langmuir model, m20-STV-0.6 GF had limited specificity for Cd(II) adsorption, with the adsorption capacity reaching 78% at a Cd(II) concentration of 5 mg/dm^3^ [151]. Mark et al. placed GF in a filter to adsorb trace Pb, Ag, and Ni metals under close to neutral and weakly acidic conditions. At low ion concentrations, the adsorption capacity of the GF was the highest. Pb and Ag were completely adsorbed, and Ni partially adsorbed on the GF, and the concentration of the Ag solution then increased. The GF material showed an adsorption limit of 4600 μg/g (43 μmol/g) [152].

#### 4.1.3. Supported Catalysts

MGF cotton has an independent structure, and it has been used as the carrier for photocatalytic and catalytic oxidation-supported catalysts [77]. Aminian et al. used GF cotton partially coated with an epoxy-resin layer as a carrier for titanium dioxide (TiO_2_) photocatalyst nanoparticles. TiO_2_ adsorbed on the epoxy groups of the GF matrix and resin layer (Figure 21D), and the photocatalytic performance of the fiber coated with TiO_2_ nanoparticles was tested for ammonia degradation. The degradation rate of the preheated fiber was approximately half of that of the unheated fiber, which was attributed to the removal of the epoxy groups of the epoxy resin on the preheated GF surface. Thus, the concentration of TiO_2_ on the GF surface decreased (Figure 21A–C) [82]. Sayaka et al. prepared a TiO_2_ porous GF-cloth composite by combining GF cloth with the TiO_2_ photocatalyst and tested the ability of the composite to remove and photocatalytically degrade 2-propanol gas. The porous material showed a high adsorption capacity for 2-propanol (Figure 21E). 2-propanol aggregated on the surface of the porous material, and photocatalytic oxidation of 2-propanol to acetone occurred until complete decomposition to CO_2_. Porous materials are a strong candidate for the degradation of gaseous organic pollutants [81]. Using flexible GF with a CuO coating as an adsorbent and a photocatalyst, Avila-Lopez et al. designed an efficient hybrid material with dual functions of capturing and photoconverting CO_2_ molecules. The material showed excellent CO_2_ adsorption efficiency. Under visible light-emitting-diode light, CO_2_ could be converted to solar fuel (CH_3_OH and HCOH) (Figure 21G). As the carrier of CuO, the GF improved CO_2_ capture and photocatalytic conversion to 11 times that of other materials [80]. Wei et al. loaded novel BiOI/BiOBr/MoS_2_ photocatalyst powder on GF to form the BiOI/BiOBr/MoS_2_–GF composite material, which was used for photocatalytic degradation of ammonia nitrogen wastewater. After four repeated experiments, the degradation rate of the wastewater by the composite material reached 74.1%. Thus, the BiOI/BiOBr/MoS_2_–GF composite is a photocatalytic material with economic value [101]. Liu et al. prepared a series of Ce/Mn-modified GF catalysts with different Ce/Mn(*x*) contents using the GF as a catalyst carrier and CeO_2_ and MnO*_x_* as active substances and studied the removal efficiency and catalytic oxidation degradation of printing and dyeing waste gas. When the O_2_ concentration was 6%, the content of chemisorbed oxygen was the highest, the efficiency of Ce/Mn-modified GF (1:5) catalytic combustion of printing and dyeing waste gas reached 90.2%, and the catalytic activity was the highest with the best stability [79].

#### 4.1.4. Gas Storage

MGF can act as a gas-storage medium based on its interaction with gas molecules. The chemical properties of MGF have comparatively higher controllability. Due to the high gas selectivity, the microporous system rather than the specific surface area is required to achieve the molecular sieve effect [153]. Through the modification of the fiber material, such as the introduction of functional groups and the interaction with the target gas, so as to achieve the adsorption purpose.

Im et al. artificially prepared fluorinated electrospun GF cotton (F-ESACF) as a storage medium for natural gas (CH_4_). The optimized storage system showed high storage capacity, fast storage and release kinetics, and high recyclability. Compared with nonfluorinated ESACF-3 and ESACF-5, fluorinated F-ESACF-3 and F-ESACF-5 showed higher methane-absorption capacities of approximately 20% and 14%, respectively, and high desorption performance of 97% [154]. Larasati used GF loaded with zeolite crystals as a CO_2_- and H_2_-storage medium. The zeolite-loaded GF showed spontaneous storage of CO_2_ and H_2_ with fast storage and release kinetics and efficient recycling. At low temperatures of 30, 40, and 50 °C, the adsorption capacities of CO_2_ were 24.6%, 15.3%, and 12.4% (w), respectively. The adsorption capacities of H_2_ at 30, 40, and 50 °C were 4.9%, 3.1%, and 2.3% (w), respectively, while the desorption capacities were 73.6%, 80.5%, and 92.5% (w), respectively. Therefore, zeolite-loaded GF is a promising storage material for CO_2_ and H_2_ [155].

Although MGF is widely used as an adsorbent, efforts are still being made to develop new adsorption properties. The development of GF with new functions can advance the field of adsorption. Because the adsorption performance is strongly dependent on the surface chemistry of the adsorbent, surface modification of the adsorbent has become a key issue in the development of unique adsorbents [125].

### 4.2. Composite Reinforcement Materials

Fibers are usually used as reinforcement materials for many polymer products [156,157,158]. GF-reinforced polymers (GFRPs) are low-cost and easy to process. GFRPs show high strength, low density and have good heat and sound insulation performance [159,160,161,162,163,164,165,166], overcoming the limitations of the individual materials and improving the comprehensive performance of the material. However, a large amount of GFRP waste is also produced, causing a substantial waste of resources, and recycling and utilization of the waste materials is a new challenge [167]. GF composite reinforcement materials are typically classified as epoxy-resin/fiber [168,169,170,171], thermoplastic/fiber (polypropylene) [172], natural-fiber/fiber [173,174], and man-made fiber/fiber reinforcement materials [175]. They can be used to strengthen fishing nets [176], concrete [177,178,179,180,181,182], guardrail systems [183], electricity poles [184], transportation pipelines [185], aerospace materials [186], and high-rise building materials [187,188].

For parts design and manufacturing of polymer/fiber composites, the interfacial adhesion is the main obstacle to obtaining excellent composite material performance [189]. The adhesion degree affects the material properties of the interface, and it depends on the nature, physical chemistry, form, and thermodynamic compatibility of the materials. A functional sandwich can be introduced between the two materials to improve the adhesion between polymers and fibers.

Fiber bridging is a toughening mechanism that can reduce crack propagation and enhance the interlaminar strength and toughness of composites. Daan reported that plasma-processed FGF, such as cotton, has significant moisture characteristics, and it can be used in other composite materials as well as in weak planes between plies of other composites. That is, a local weak plane is introduced without using any physical barrier to reduce delamination, a common damage mechanism in composites, by maintaining a certain interlaminar strength [129].

#### 4.2.1. Resin/Fiber-Reinforced Materials

Cech et al. synthesized fiber-reinforced polyester resin composites using GF as the matrix, plasma polymer films (PP-HMDSO and PP-VTES), and silane polymer films (PC-VTES) deposited on the fiber surface as the intermediate layer and tested the interfacial shear strength of the GF/polyester interface. The interfacial shear strength (IFSS) values of the PP-VTES- and PC-VTES-modified glass/polyester composites were 110% higher than that of the untreated glass/polyester composite, and the performance was comparatively better (Figure 4C,D) [62]. Takizawa stacked and molded BNNT/toluene-solution-treated GF epoxy-resin matrix composites. Compared with that of the unmodified composite, the thermal conductivity of the BNNT/toluene/GF composite increased by 90% to 1.2 W/(m·K) without affecting the electrical insulation of the composite, and it also showed higher ductility [88]. Tamrakar et al. modified an epoxy-resin matrix with PEI-CNTs to enhance the mechanical properties of the epoxy resin (Figure 8D). The IFSS was proportional to the thickness of the coating. The combination of a 2 μm-thick PEI-CNT coating (SG-1-15-15m) and unmodified epoxy resin showed the highest IFSS (75.5 MP), which was 58% higher than that of the matrix [95]. Chen et al. synthesized GO-GF/epoxy-resin composites and measured the interlaminar shear strength (ILSS) of the composites (Figure 15B). The concentration of the GO solution ranged from 1.0 to 2.0 mg/mL (1.0, 1.5, and 2.0 mg/mL). When the concentration of GO was 1.5 mg/mL, the ILSS of the composite reached the maximum value. In addition, the ILSS of GO-G-GF (chemical grafting method) was higher than that of GO-C-GF (electrostatic assembly strategy method), and the GO layer improved the interfacial adhesion between the GF and polymer matrix [65,90]. Sarr et al. compounded CNT-GF with an epoxy resin to form the GF-CNT/epoxy-resin composite, which improved the mechanical properties of the GF/epoxy-resin composite material. Through the mechanism of interface peeling, mechanical interlocking, and the resin toughness of the CNF bridge, the IFSS increased by a maximum of 78% and the bending strength increased by 20% [67].

#### 4.2.2. Thermoplastic/Fiber-Reinforced Materials

Thermoplastic/fiber composites are composed of a thermoplastic matrix and a GF-reinforced material. Commonly used thermoplastics are polypropylene [164,190], polytetrafluoroethylene [189,191], polyvinyl chloride [192], nylon (PA6) [193], and PET [194]. Fang et al. prepared isometric polypropylene (IPP)-modified GF micro-composites. They combined PEI-CNT-modified GF (PEI-CNT-GF) and PEI-GO-modified GF (PEI-GO-GF) with IPP plastic, forming a dense crystallized structure on the interface between the two components (Figure 16D–F). IPP/PEI-CNT-GF and IPP/PEI-GO-GF showed good interface adhesion. Compared with the IFSS values of PEI-CNT-GF and PEI-GO-GF, the IFSS values of IPP/PEI-CNT-GF and IPP/PEI-GO-GF increased by 39.5% and 34.5%, respectively (Figure 16G), and thus the interface performance significantly improved [120]. Hamid et al. performed C–H insertion crosslinking modification to improve the mechanical properties of GF-reinforced polypropylene. The stress–strain curves showed that the maximum stress of the 14.0 vol% GF was twice that of the 4.3 vol% GF (35.0 MPa). The maximum strain of the 14 vol% GF was approximately half of that of the 4.3 vol% GF (0.595), and a higher volume fraction led to higher overall strength [61]. Guo et al. used nickel-coated GF as a conductive filler to prepare a polypropylene/nickel-coated GF composite, and the conductive filler improved the conductive properties of the GF [77]. On this basis, they introduced the nucleating agent dibenzyl sorbitol to promote the formation of a complete conductive network in the nickel-coated GF. Thereby, the conductivity and mechanical properties of the polypropylene/nickel-plated GF composite were further improved, providing a feasible idea for preparing high-performance conductive composites [195]. Fang et al. used a simple, environmentally friendly, and effective method to improve the interface properties of GF and PA6 matrix composites and used PEI-CNT-GF to form PA6/PEI-CNT-GF composites. Hydrogen-bond interaction, mechanical interlocking, and the crystalline structure of the bridge model promoted the close bonding of PEI-CNT-GF with the PA6 plastic at the interface. The PA6/PEI-CNT-GF composite showed the best mechanical properties (Figure 16C). Compared with those of PA6/GF, the tensile strength, bending strength, and elongation at break of PA6/PEI-CNT-GF increased by approximately 7.5%, 6.9%, and 17.5%, respectively, showing the broad application prospects for interface modification of thermoplastic composites [119]. Xiao et al. used BP and RP to enhance the flame retardancy of PET-modified GF (PET/GF) and tested the thermal stability and fire resistance of BP/PET/GF and RP/PET/GF. Addition of BP and RP improved the flame-retardant performance of PET/GF, but the flame-retardant efficiency of BP on PET/GF was better than that of RP on PET/GF, which was attributed to the dispersity and carbon catalytic properties of BP. Good dispersity obstructs heat transfer at the initial stage of combustion, and the carbon catalyst promotes formation of a dense carbon layer in the middle stage of combustion. The “wick effect” of GF is inhibited, which broadens the application field of GF-reinforced polymers [25,40]. Li et al. prepared PTFE/FGF microwave composites by mixing FGF with PTFE and studied the mechanical properties, density, and hygroscopicity of the composites (Figure 17D,E). The tensile strength (38.53 MPa) and elongation (226.82%) of the PTFE/FGF composite were significantly higher than those of the PTFE/MGF (34.73 MPa and 179.50%, respectively) and PTFE/GF (34.82 MPa and 174.33%, respectively) composites. In addition, the density (2.174 g cm^−3^) and hygroscopicity (0.008%) of the PTFE/FGF composite were slightly improved, which improved the comprehensive properties of the composite [127]. Zhou et al. fabricated PDA–PTFE/GF composite materials. The composite materials showed strong hydrophilicity, and the coating showed good chemical and mechanical stability. Compared with the PDA coating, the PDA coating formed using NaIO_4_ as an oxidant showed better hydrophilicity and stability [1].

#### 4.2.3. Natural Fiber/Fiber-Reinforced Materials

Compared with resins and thermoplastics, natural fibers are rich in variety, environmentally friendly, and show excellent biodegradability and reproducibility [196,197]. Despite the abovementioned advantages, natural fibers still have some disadvantages, such as limited thermal stability, high hygroscopicity, low dispersion, and poor interface adhesion between the natural fibers and matrix [198,199,200,201], so natural fibers are not widely used in GFRPs [202].

Jing et al. used S-GF and GO-GF to enhance polylactic acid (PLA) and studied the mechanical and thermodynamic properties of S-GF–PLA and GO-GF–PLA composites. The addition of S-GF significantly improved the mechanical properties and toughness, which resulted from the optimal length of the retained fibers. Crystallization of GO-GF on the composite interface increased Young’s modulus and further enhanced the properties of the GO-GF–PLA composite [89]. Ramesh et al. developed sisal/jute-GF-reinforced polyester composites. The composites showed good tensile strength, flexural strength, and impact strength, and they could be used as an alternative to GF-reinforced polymer composites [203].

### 4.3. Biomedical Science

MGF has great potential in bacteriostasis, biological detection, and repair, and it can be applied to disinfection and sterilization [32,74,103,204], clinical diagnosis [29], and chemotherapy [205,206]. However, the biotoxicity and biocompatibility of MGF need to be further investigated.

Li et al. reported that the three components of the GO/ZnO/Cu_2_O/GF composite coating could synergistically enhance the antibacterial performance. *Staphylococcus aureus* and *Escherichia coli* were used as experimental objects to test the antibacterial activity of the GO/ZnO/Cu_2_O/GF hybrid material. The coating showed a certain degree of antibacterial performance against the two strains, and the antibacterial effect against *S. aureus* was better than that against *E. coli* [100]. Song et al. described an antibody-coupled GFM platform to detect the cardiac calcium protein (cTnT) in the blood (Figure 13B). This method can be used for linear quantification of cTnT in the range of 1.0–120 pg/mL within 30 min at 25 °C. It shows high sensitivity, high accuracy, and high specificity, providing a feasible method for the detection of biomolecular cTnT [69]. Fang et al. developed a new microfluidic system by combining GF as a medium and a paper-based microfluidic system. The system showed high micropattern resolution, fast flow rate, and high loading capacity, and it can detect protein, glucose, ketone bodies, nitrite, and the pH value in urine in a microarray format [207]. Liverani evaluated the wound closure efficiency of copper-doped GF by the scratch test (Figure 13C). Without affecting keratinocyte formation and migration, sustained release of Cu^2+^ inhibited mineralization of fibers in simulated body fluids and achieved scratch wound closure within 48 h [113]. Vallittu et al. applied GF-reinforced resin as an oral implant in dental materials, prosthodontics, periodontal treatment, and orthodontics. The reinforced material resisted the mechanical stress of the chewing system and showed good fatigue resistance and toughness [208,209].

### 4.4. Other Applications

In addition to the above application fields, the application of MGF has been extended to optical fibers [210,211,212,213,214,215], lithium-oxygen batteries [216,217], and strain sensors [218,219,220,221].

Saito et al. prepared PTFE-clad As–S GF and applied it to infrared fibers. The transmission range of the fiber was 1–7 µm, and the minimum optical loss was as low as 0.15 dB/m, which was lower than the lowest optical loss of other fiber core-clad As–S fiber (0.197 dB/m). The absorption and scattering loss of the As–S fiber were also reduced [211]. Woo applied a double film composed of a lithium-ion-conductive ceramic film and a GF film to a lithium-oxygen battery. The battery showed high ionic conductivity of 8.1 × 10^−4^ S/cm, no capacity attenuation during 70 charge and discharge cycles, and the increases of the electrolyte resistance and interface resistance were relatively small. The performance was superior to the performance using the composite polymer membrane or GFM alone [216]. Moriche et al. applied GNP–GF and NH_2_-GNP–GF composites to strain monitoring and obtained high sensitivity values under tensile loads, reaching the order of 840 to 16,400 [93]. Sebastian et al. integrated CNT-GF as a sensor into composite materials and used them as a fuzzy fiber strain gauge to detect structural integrity by sensing multiple strain tasks in composite structures, including the strain, the temperature, and degradation. The strain gauge showed similar sensitivity to traditional strain gauges [218].

## 5. Summary and Outlook

The modification methods, properties, and applications of MGF have been reviewed. Based on the chemical reactivity of the Si–O bond and its subsequent derivative reactions, a variety of functional groups can be grafted on the GF surface, which provides a general route for the preparation of various GF derivatives. In addition, the unique properties of MGF have been gradually developed, and MGF shows great application prospects in adsorption, composite reinforcement, and biomedical fields.

Although remarkable progress has been made in MGF, from preparation to application, there are still some difficult problems that need to be solved, including (I) fine structural design, (II) high-quality green preparation, (III) efficient adsorption performance, and (IV) recycling of waste glass/waste GF.

I. Fine structural engineering is the key factor in optimizing the physical and chemical properties of GF. Refined structural engineering distinguishes the specific contributions of GFs with different structures in their performance. This provides guidance for the design of specific functionally graded materials and ultimately achieving customized MGF products to meet the needs of different application fields, as well as providing guidance for targeted, precise modification of other materials.
II. Development of new GF modification methods is required. Using the MGF surface active group, ingenious three-dimensional symmetric configurations can be designed to realize composites with other functional materials and develop more green and environmentally friendly high-performance materials.
III. Application of MGF in the field of adsorption needs to be expanded. In some gaseous media, especially aerosols, under very low detection conditions, combined with the special sites of MGF, new strong adsorption forces, adsorbent enrichment storage, directional transfer, and desorption need to be investigated to achieve reuse and the perfect combination of MGF and environmental media.
IV. A recycling route to add value, recycle waste glass as a raw material for GF preparation, and reuse the used MGF needs to be developed to expand production and reduce the cost of GF to realize a sustainable circular supply chain and circular economy.

## Data Availability

Not applicable.
